# Long-term benefits of dapagliflozin on renal outcomes of type 2 diabetes under routine care: a comparative effectiveness study on propensity score matched cohorts at low renal risk

**DOI:** 10.1016/j.lanepe.2024.100847

**Published:** 2024-02-01

**Authors:** Gian Paolo Fadini, Enrico Longato, Mario Luca Morieri, Stefano Del Prato, Angelo Avogaro, Anna Solini, DARWIN-Renal Study Investigators, Mariella Baldassarre, Agostino Consoli, Sara Morganet, Antonella Zugaro, Marco Giorgio Baroni, Francesco Andreozzi, Adriano Gatti, Adriano Gatti, Stefano De Riu, Andrea Del Buono, Raffaella Aldigeri, Riccardo Bonadonna, Alessandra Dei Cas, Angela Vazzana, Monica Antonini, Valentina Moretti, Patrizia Li Volsi, Miranda Cesare, Giorgio Zanette, Silvia Carletti, Paola D'Angelo, Gaetano Leto, Frida Leonetti, Luca D'Onofrio, Ernesto Maddaloni, Raffaella Buzzetti, Simona Frontoni, Giselle Cavallo, Susanna Morano, Tiziana Filardi, Umberto Capece, Andrea Giaccari, Antonio C. Bossi, Giancarla Meregalli, Fabrizio Querci, Alessia Gaglio, Veronica Resi, Emanuela Orsi, Stefano Fazion, Ivano G. Franzetti, Cesare Berra, Silvia Manfrini, Gabriella Garrapa, Giulio Lucarelli, Lara Riccialdelli, Elena Tortato, Marco Zavattaro, Gianluca Aimaretti, Franco Cavalot, Guglielmo Beccuti, Fabio Broglio, Bruno Fattor, Giuliana Cazzetta, Olga Lamacchia, Anna Rauseo, Salvatore De Cosmo, Rosella Cau, Mariangela Ghiani, Antonino Di Benedetto, Antonino Di Pino, Salvatore Piro, Francesco Purrello, Lucia Frittitta, Agostino Milluzzo, Giuseppina Russo

**Affiliations:** a Division of Metabolic Diseases, Department of Medicine, University of Padova, 35128 Padova, Italy; b Laboratory of Experimental Diabetology, Veneto Institute of Molecular Medicine, 35128 Padova, Italy; c Department of Information Engineering, University of Padova, 35100 Padua, Italy; d Department of Clinical & Experimental Medicine, University of Pisa and Sant’Anna School of Advanced Studies, 56126 Pisa, Italy; e Department of Surgical, Medical, Molecular and Critical Area Pathology, University of Pisa, Italy

**Keywords:** Type 2 diabetes, Chronic kidney disease, SGLT2 inhibitors, Prevention, Observational

## Abstract

**Background:**

Despite the overall improvement in care, people with type 2 diabetes (T2D) experience an excess risk of end-stage kidney disease. We evaluated the long-term effectiveness of dapagliflozin on kidney function and albuminuria in patients with T2D.

**Methods:**

We included patients with T2D who initiated dapagliflozin or comparators from 2015 to 2020. Propensity score matching (PSM) was performed to balance the two groups. The primary endpoint was the change in estimated glomerular filtration rate (eGFR) from baseline to the end of observation. Secondary endpoints included changes in albuminuria and loss of kidney function.

**Findings:**

We analysed two matched groups of 6197 patients each. The comparator group included DPP-4 inhibitors (40%), GLP-1RA (22.3%), sulphonylureas (16.1%), pioglitazone (8%), metformin (5.8%), or acarbose (4%). Only 6.4% had baseline eGFR <60 ml/min/1.73 m^2^ and 15% had UACR >30 mg/g. During a mean follow-up of 2.5 year, eGFR declined significantly less in the dapagliflozin vs comparator group by 1.81 ml/min/1.73 m^2^ (95% C.I. from 1.13 to 2.48; p < 0.0001). The mean eGFR slope was significantly less negative in the dapagliflozin group by 0.67 ml/min/1.73 m^2^/year (95% C.I. from 0.47 to 0.88; p < 0.0001). Albuminuria declined significantly in new-users of dapagliflozin within 6 months and remained on average 44.3 mg/g lower (95% C.I. from −66.9 to −21.7; p < 0.0001) than in new-users of comparators. New-users of dapagliflozin had significantly lower rates of new-onset CKD, loss of kidney function, and a composite renal outcome. Results were confirmed for all SGLT2 inhibitors, in patients without baseline CKD, and when GLP-1RA were excluded from comparators.

**Interpretation:**

Initiating dapagliflozin improved kidney function outcomes and albuminuria in patients with T2D and a low renal risk.

**Funding:**

Funded by the Italian Diabetes Society and partly supported by a grant from 10.13039/100004325AstraZeneca.

**Research in context**

**Evidence before this study**

In randomized controlled trials (RCTs), sodium glucose co-transporter-2 inhibitors (SGLT2i) reduced the risk of a composite kidney outcome and preserved kidney function over time. Subsequent trials conducted in patients with stage II-III CKD and micro-macroalbuminuria, with or without diabetes, demonstrated that SGLT2i reduced the risk of ESKD, heart failure, and cardiovascular events. We searched the literature for real-world observational studies reporting the effects of SGLT2i on kidney outcomes in large populations. The search strategy was: ("real-world" or "observational") and ("empagliflozin" or "dapagliflozin" or "canagliflozin" or "ertugliflozin" or "SGLT2") and ("kidney" or "renal") and ("eGFR" or "albuminuria" or "AER" or "UACR" or "UAER"). We found that several real-world studies have confirmed the ability of SGLT2i to protect from adverse kidney outcomes, but methodological issues make interpretation of the results of such studies not always straightforward. Channelling bias, time-lag bias, conditioning on the future, database heterogeneity, linearity assumptions on eGFR trends and duration of observation remain to be addressed in order to provide more robust evidence. In addition, large observational studies reporting eGFR-based endpoints often lack information on albuminuria and other intermediate endpoints (e.g., HbA1c, body weight, and blood pressure).

**Added value of this study**

We designed a multi-center retrospective study to compare the effectiveness of the SGLT2i dapagliflozin vs non-SGLT2i non-insulin glucose-lowering medication on kidney endpoints based on eGFR and albuminuria, as well as on intermediate endpoints. We aimed to limit as much as possible the biases due to confounding by indication, conditioning on the future, and non-linearity of eGFR change.

In a large nation-level observational cohort of outpatients with type 2 diabetes and a low prevalence of kidney disease at baseline, new users of dapagliflozin, as compared to new users of other glucose lowering medications, were protected from the decline in kidney function, the rise in albuminuria, and the occurrence of a composite adverse kidney outcome. Robust evidence of renal protection by dapagliflozin was obtained despite less reduction in HbA1c and marginal differences in body weight and blood pressure.

**Implications of all the available evidence**

The strong efficacy of SGLT2i against kidney disease demonstrated in randomized trials has the potential to change the epidemiology of chronic kidney disease in the future years. From a public health perspective, it is important that such benefits are confirmed by high-quality data from the routine care setting, especially for low-risk populations. For the first time, we show the effectiveness of SGLT2i on kidney outcomes, including the loss of kidney function and albuminuria, and on intermediate outcomes relevant to diabetes management. This retrospective evaluation adds to the existing evidence and further supports the large-scale adoption of SGLT2i to prevent new-onset and worsening kidney disease in diabetes.

## Introduction

Approximately 1 in 3 adults with diabetes has chronic kidney disease (CKD). Diabetes accelerates the progression to end-stage kidney disease (ESKD), which represents a serious burden to the person, his/her families, and the healthcare system. While multifactorial intervention for the management of type 2 diabetes (T2D) has improved cardiovascular outcomes, the incidence of ESKD and the need for dialysis or transplantation have not decreased.^1^^,^^2^ Moreover, diabetic kidney disease shortens life expectancy by up to 16 years.^3^

In secondary analyses of large cardiovascular outcome trials, sodium glucose co-transporter-2 inhibitors (SGLT2i) reduced the risk of a composite kidney outcome and preserved kidney function over time.^4^ Subsequent trials enrolled patients with stage II–III CKD and micro-macroalbuminuria with or without diabetes, and demonstrated that SGLT2i reduced the risk of ESKD, heart failure, and cardiovascular events.^5^, ^6^, ^7^ Such degree of renal protection has not been demonstrated for other diabetes medications: while GLP-1 receptor agonists (GLP-1RA) protect from cardiovascular events, they have shown modest renal protective effects on albuminuria and on the decline in kidney function.^8^, ^9^, ^10^

Trial populations consisted of patients with high cardio-renal risk and were not representative of the majority of T2D with impaired renal function. In fact, the normoalbuminuric phenotype is the prevailing form of renal impairment in T2D,^11^ occurring in high-risk individuals who progress to ESKD and have high cardiovascular risk.^12^ In addition, it remains unclear whether SGLT2i protect from adverse kidney outcomes in primary renal prevention or in populations at low baseline renal risk.

While randomized controlled trials typically enrol highly selected populations, studies using real-world data are increasingly valued as they can better address the patient population receiving treatment under routine care, outside the experimental trial design. Several real-world studies have confirmed the effectiveness of SGLT2i in treating diabetes and protecting against cardiovascular disease and mortality.^13^ Despite observational research have confirmed the ability of SGLT2i to protect from adverse kidney outcomes,^14^ some issues in the real-world studies on the renal effects of SGLT2i make data interpretation not always straightforward. Channelling bias, time-lag bias, conditioning on the future, database heterogeneity, linearity assumptions on eGFR trends, and duration of observation are critical issues that undermine robustness of such studies.^15^ These challenges have been considered and addressed in the DApagliflozin Real-World EvIdeNce (DARWIN)-Renal, a study commissioned by the Italian Diabetes Society with the aim of expanding our knowledge on the long-term effects of SGLT2i on renal function in a large cohort of individuals with T2D. We compared kidney outcomes of patients who initiated dapagliflozin or other diabetes medications other than SGLT2i and insulin. Despite the comparator group included drugs exerting some renal protection, like GLP-1RA, we hypothesized that benefits of the SGLT2i dapagliflozin against the loss of kidney function could be confirmed in patients with low renal risk.

## Methods

### Study design and objective

DARWIN-Renal was a multicentre retrospective comparative effectiveness study conducted by the Italian Diabetes Society at 50 diabetes specialist care clinics in Italy. The rationale and design of the study have been described in a prior publication.^15^ The conduct and reporting of the study followed the STROBE checklist,^16^ including the proposed addendum for the comparison of matched cohorts.^17^ The general objective was to compare kidney outcomes of patients who initiated dapagliflozin vs other glucose lowering medications (GLM). During the study period, in Italy, SGLT2i could be prescribed only by diabetes specialists. Data were collected at all centres from the same electronic health record system (Smart Digital Clinic, Meteda Srl, San Benedetto del Tronto, Italy) using an automated data extraction software. Patients’ records were anonymised according to local and international standards. The study was conducted in agreement with the declaration of Helsinki and the protocol was approved by the Ethics Committee of the coordinating centre (University Hospital of Pisa, Italy) and all participating centres. Based on the national regulation on retrospective studies using anonymised data, patient’s informed consent was waived. The study was funded by the Italian Diabetes Society and partly supported by a grant from AstraZeneca.

### Definition of cohorts and endpoints

We defined two groups of patients based on their newly initiated GLM between 01/01/2015 and 30/09/2020. The main group of interest consisted of patients who initiated dapagliflozin. The control group consisted of patients who initiated any other GLM, excluding insulin and other SGLT2i. Such comparator drugs included: metformin, DPP-4 inhibitors, GLP-1 receptor agonists (GLP-1RA), sulphonylurea/glinides (grouped together), pioglitazone, and acarbose. The index date for each patient was set as the date when patients were prescribed for the first time a new therapy with either dapagliflozin or a comparator. Patients could be included if they were aged 18–80, had type 2 diabetes since at least one year, had initiated for the first time (as evident from the database) the GLM of interest, and had available information on renal outcomes. The exclusion criteria were: other forms of diabetes; age <18 or >80; previous therapy with another SGLT2i in the 12 months prior to the index date; CKD stage V or dialysis. Note that, for most of the observation period, initiating dapagliflozin was contraindicated in patients with eGFR <60 ml/min/1.73 m^2^ and is still not recommended with eGFR <25 ml/min/1.73 m^2^.

The primary analysis consisted in the comparison of kidney outcomes between patients who initiated dapagliflozin and those who initiated comparator drugs. The primary outcome was the change over time in eGFR, calculated according to the CKD-EPI equation.^18^

Secondary outcomes included: evaluation of total and chronic (from 6 months on) eGFR slopes; the change over time in UACR, HbA1c, body weight, and blood pressure; new-onset CKD (defined as the occurrence of two eGFR values <60 ml/min/1.73 m^2^ at least 90 days apart, among those who had a baseline eGFR >60 ml/min/1.73 m^2^); the change (worsening) in CKD class (stage I eGFR ≥90; stage II 60–90; stage IIIa 45–60; stage IIIb 30–45; stage VI 15–30; stage V <15 ml/min/1.73 m^2^); substantial loss of kidney function (defined as a reduction of eGFR of 40% or greater relative to baseline value); doubling of serum creatinine (equal to a reduction of eGFR of 57% or greater relative to baseline value); ESKD (defined as a confirmed eGFR <15 ml/min/1.73 m^2^ in at least two occasions at least 90 days apart); initiation of dialysis; albuminuria class improvement.

### Data collection

For all patients, the following information were collected from the electronic chart at the index date, with a grace period of −90 days: demographics (age, sex, diabetes duration); anthropometrics (height, weight, BMI, waist circumference); systolic and diastolic blood pressure; laboratory data (fasting glucose, HbA1c, total cholesterol, HDL cholesterol, triglycerides, calculated LDL cholesterol; serum creatinine for the calculation of the eGFR; albumin excretion rate normalised to the mg/g of urinary creatinine as previously described^19^); presence or absence of chronic diabetic complications as recorded in the electronic health records; and background therapy for diabetes and for the control of cardiovascular risk factors. Details on the data collection methodology for the DARWIN study series have been described previously.^20^ We also collected pre-index date eGFR values in order to compute the baseline eGFR slope.

The same set of information was collected at follow-up visits after the index date. In particular, for the evaluation of endpoints, we recorded updated values of body weight, blood pressure, HbA1c, and renal function exams (eGFR and UACR).

### Sample size calculation

According to previous data from the DARWIN study series,^19^ the standard deviation of eGFR was around 15 ml/min/1.73 m^2^. With power 90% and alpha 5%, we estimated a sample size of 1184 patients/group to detect a difference in eGFR change of 2 ml/min/1.73 m^2^. Considering that up to 50% of patients in the electronic health records may not have available data for the primary endpoint, we needed to identify at least 2368 patients initiating dapagliflozin, to be matched with patients initiating comparators from an expectedly larger initial population. Given that no assumption could be made on the achievable number of patients after propensity score matching (PSM), recruitment was continued until completion of the planned number of contributing centres, even if yielding a larger final sample size.

### Statistical analysis

Continuous variables are presented as mean and standard deviation, whereas categorical variables are reported as percentages. Normality of continuous variables was verified with one-way Kolmogorov–Smirnov test and non-normal variables were log-transformed before analysis with parametric tests (log-transformed variables are shown in their original unit of measure for clarity of reading). Comparisons between two groups were performed using the Wilcoxon–Mann–Whitney test and the chi square test, as appropriate.

The change over time in continuous variables (including the analysis of eGFR for the primary outcome) was compared between the two groups using the mixed model for repeated measures (MMRM). eGFR, UACR, HbA1c, body weight, and blood pressure were used as the dependent variables. The testing for UACR was performed with log10 values but data are shown in the original unit of measure. Treatment group (dapagliflozin vs comparators), time, and the group by time interaction were entered as fixed effects (along with the intercept). The heterogeneous compound symmetry was chosen as the variance structure. The output of the MMRM were the marginal means in each group and the mean difference between groups, and their standard errors. Rates of occurrence of categorical outcomes were compared between the two groups using the Cox proportional hazards model, reporting hazard ratios (HR) and 95% confidence intervals (C.I.). The proportional hazards assumption was verified by visual inspection and Schoenfeld residuals. To address confounding by indication, we performed PSM of patients who initiated dapagliflozin and those who initiated a comparator. Propensity scores were calculated with a logistic regression model where treatment was the dependent variable and covariates were those listed in Table 1, chosen using the modified disjunctive cause criterion.^21^ Patients in the two groups were matched 1:1 with a caliper of 0.1 pooled standard deviations using nearest neighbour without replacement. Between-group balance before and after PSM was evaluated by calculating the standardized mean difference (SMD). SMD values <0.1 were considered to be indicative of a good balance. Success of PSM was defined as the absence of residual imbalance (SMD ≥0.1) in all the variables listed in Table 1. Given than PSM requires a complete case dataset and some data were missing in the database (ranging from 5.2% for baseline HbA1c to 53% for albuminuria) we performed multiple imputation by chained equations (MICE) to obtain 10 imputed datasets. Imputation was performed on the same variables used for PSM, without a priori constraints, and setting the maximum number of iterations to 20.

**Table 1:** Characteristics of the matched cohorts in the first imputed dataset.

	Before PSM			After PSM		
	Dapagliflozin	Comparators	SMD	Dapagliflozin	Comparators	SMD
**Number**	7588	27,717		6200	6200	
**Demographics**						
Sex male, n (%)	4575 (60.3)	16,796 (60.6)	<0.01	3808 (61.4)	3789 (61.1)	<0.01
Age, years	60.9 (9.0)	63.6 (8.8)	0.30	61.0 (9.0)	61.3 (9.6)	0.03
Diabetes duration, years	11.6 (8.4)	10.6 (7.9)	0.12	10.6 (7.9)	10.4 (8.3)	0.02
**Anthropometrics**						
Weight, kg	88.9 (18.4)	83.8 (17.8)	0.28	88.0 (18.0)	87.7 (19.0)	0.01
Height, cm	167.2 (9.7)	166.6 (9.7)	0.06	167.3 (9.6)	167.3 (9.7)	<0.01
Body mass index, kg/m^2^	31.8 (5.8)	30.2 (5.8)	0.27	31.4 (5.7)	31.3 (6.2)	<0.01
Waist, cm	109.3 (13.4)	105.5 (13.4)	0.28	108.3 (13.3)	108.0 (14.1)	0.02
**Risk factors and laboratory**						
Systolic blood pressure, mm Hg	137.8 (18.8)	136.4 (18.6)	0.08	137.1 (18.7)	137.2 (18.6)	<0.01
Diastolic blood pressure, mm Hg	79.3 (10.2)	78.2 (9.9)	0.11	79.4 (10.2)	79.4 (10.2)	<0.01
Fasting plasma glucose, mg/dl	173.6 (57.2)	159.6 (48.0)	0.28	169.0 (53.8)	168.4 (54.5)	0.01
HbA1c, %	8.4 (1.5)	7.8 (1.2)	0.44	8.2 (1.4)	8.2 (1.5)	0.02
Total cholesterol, mg/dl	174.2 (43.7)	172.5 (42.8)	0.04	173.7 (43.5)	173.9 (43.4)	<0.01
HDL cholesterol, mg/dl	46.7 (14.6)	48.4 (15.2)	0.12	46.7 (14.6)	47.1 (14.4)	0.02
LDL cholesterol, mg/dl	94.9 (36.2)	94.3 (35.7)	0.02	94.7 (36.0)	95.2 (35.6)	0.01
Triglycerides, mg/dl	143 (102–200)	132 (95–184)	0.14	141 (101–198)	139 (99–195)	0.03
eGFR (ml/min/1.73 m^2^)	87.7 (16.7)	80.7 (21.1)	0.35	87.7 (16.8)	87.4 (17.9)	0.01
Albumin excretion rate, mg/g	10.5 (5.3–24.9)	10.6 (5.3–25.0)	<0.01	10.4 (5.1–22.5)	10.5 (5.3–24.3)	<0.01
Normoalbuminuria	8.0 (4.5–15.0)	8.3 (4.50–14.9)	<0.01	8.0 (4.5–15.0)	8.4 (4.5–15.0)	<0.01
Microalbuminuria	71.5 (45.0–127.4)	68.0 (43.9–124.5)	0.06	72.1 (45.2–126.4)	69.1 (44.5–126.0)	0.04
Macroalbuminuria	654.0 (431.5–1189.5)	690.1 (415.7–1349)	0.07	648.0 (429.0–1119.0)	631.0 (411–1239)	0.02
eGFR slope (ml/min/1.73 m^2^/year)	−0.6 (2.4)	−1.1 (2.4)	0.19	−0.7 (2.1)	−0.7 (2.1)	0.02
**Complications**						
Chronic kidney disease, n (%)	1535 (20.2)	7750 (28.0)	0.18	1219 (19.7)	1246 (20.1)	0.01
EGFR <60 ml/min/1.73 m^2^, n (%)	416 (5.5)	4651 (16.8)	0.33	394 (6.4)	389 (6.3)	<0.01
UACR >30 mg/g, n (%)	1229 (16.2)	4182 (15.1)	0.03	952 (15.4)	928 (15.0)	0.01
Diabetic retinopathy, n (%)	1399 (18.4)	3723 (13.4)	0.14	927 (15.0)	919 (14.8)	<0.01
Diabetic macular edema, n (%)	200 (2.6)	507 (1.8)	0.06	127 (2.0)	140 (2.3)	0.01
Stroke/TIA, n (%)	106 (1.4)	439 (1.6)	0.02	79 (1.3)	84 (1.4)	<0.01
Carotid atherosclerosis, n (%)	1397 (18.4)	5886 (21.2)	0.07	1090 (17.6)	1127 (18.2)	0.02
Ischemic heart disease, n (%)	800 (10.5)	3009 (10.9)	0.01	594 (9.6)	582 (9.4)	<0.01
Left ventricular hypertrophy, n (%)	581 (7.7)	2214 (8.0)	0.01	461 (7.4)	460 (7.4)	<0.01
Heart failure, n (%)	234 (3.1)	693 (2.5)	0.04	179 (2.9)	171 (2.8)	<0.01
Any site revascularization, n (%)	527 (6.9)	2130 (7.7)	0.03	387 (6.2)	387 (6.2)	<0.01
Microvascular complications, n (%)	2824 (37.2)	11,116 (40.1)	0.06	2119 (34.2)	2093 (33.8)	<0.01
Macrovascular complications, n (%)	2376 (31.3)	9383 (33.9)	0.05	1847 (29.8)	1826 (29.5)	<0.01
Established CVD, n (%)	975 (12.8)	3776 (13.6)	0.02	725 (11.7)	720 (11.6)	<0.01
**Glucose lowering medications**						
Metformin, n (%)	6185 (81.5)	20,890 (75.4)	0.15	5224 (84.3)	5184 (83.6)	0.02
Sulphonylurea/repaglinide, n (%)	621 (8.2)	5383 (19.4)	0.30	617 (10.0)	652 (10.5)	0.02
DPP-4 inhibitors, n (%)	108 (1.4)	3130 (11.3)	0.35	107 (1.7)	111 (1.8)	<0.01
GLP-1 receptor agonists, n (%)	203 (2.7)	1002 (3.6)	0.05	199 (3.2)	206 (3.3)	<0.01
Pioglitazone, n (%)	157 (2.1)	1231 (4.4)	0.12	150 (2.4)	155 (2.5)	<0.01
Acarbose, n (%)	61 (0.8)	223 (0.8)	<0.01	52 (0.8)	55 (0.9)	<0.01
Bolus insulin, n (%)	1943 (25.6)	682 (2.5)	0.95	563 (9.1)	598 (9.6)	0.02
Basal insulin, n (%)	3162 (41.7)	4471 (16.1)	0.64	1814 (29.3)	1746 (28.2)	0.02
**Other medications**						
Statins, n (%)	4196 (55.3)	14,960 (54.0)	0.03	3334 (53.8)	3292 (53.1)	0.01
Anti-platelet agents, n (%)	2757 (36.3)	10,444 (37.7)	0.03	2130 (34.4)	2062 (33.3)	0.02
RAS blockers, n (%)	4359 (57.4)	15,733 (56.8)	0.01	3495 (56.4)	3471 (56.0)	<0.01
Beta blockers, n (%)	2058 (27.1)	7444 (26.9)	<0.01	1643 (26.5)	1652 (26.6)	<0.01
Calcium channel inhibitors, n (%)	1579 (20.8)	5972 (21.5)	0.02	1271 (20.5)	1288 (20.8)	<0.01
Diuretics, n (%)	2125 (28.0)	8593 (31.0)	0.07	1699 (27.4)	1727 (27.9)	0.01
Anticoagulants, n (%)	142 (1.9)	859 (3.1)	0.07	124 (2.0)	120 (1.9)	<0.01

Data are presented for patients who initiated dapagliflozin or comparators before and after propensity score matching. Continuous variables are presented as mean (standard deviation) and median (IQR). Categorical variables are presented as number (percentage).

Microvascular complications included retinopathy, neuropathy, and nephropathy. Macrovascular complications included evidence of atherosclerosis in any arterial site. Established cardiovascular disease (CVD) was defined as myocardial infarction, stroke or transient ischemic attack (TIA), or prior arterial revascularization.

RAS, renin angiotensin system; SMD, standardized mean difference.

All analyses were run on each of the 10 datasets and results were then pooled using the standard approach developed by Rubin.^22^^,^^23^ Imputation was used only for matching purposes and all data being shown refer to truly available information. Imputed values were not used for outcome analysis. For the analysis of secondary endpoints, some patients with missing follow-up data had to be excluded. Persistence of a good balance in subgroups of the matched populations was verified in the same way as for the original PSM procedure. Variables that were consistently imbalanced (SMD >0.1) in at least 5 of the 10 imputed datasets were entered as covariates in the MMRM or survival analyses. As there is no consensus on whether paired or unpaired analyses should be performed after PSM,^24^^,^^25^ the comparison between matched cohorts was performed preferentially with an unpaired approach but the main findings were confirmed with the paired approach. For the MMRM, we included random effects that explicitly modelled the fact that observations in the same pair belonged to the same stratum.^26^ For the Cox model, we specified the matched pairs into the model and used a marginal survival model with robust standard errors.^27^

The primary analysis was conducted in the intention-to-treat (ITT) population, comprising all new-users of the drugs of interest who had at least one eGFR value post-index date, censored at event occurrence or last observation. We performed a sensitivity analysis on the on-treatment (OT) population, censoring patients at the time of index drug discontinuation, the event, or last observation, whichever occurred first. Drug discontinuation was defined as the first follow-up visit when the drug was no longer prescribed.

The primary endpoint was re-examined in subgroups of patients based on pre-specified clinical characteristics at baseline. Patients were divided into strata and the mean between-group difference in eGFR was calculated in each stratum and compared across strata. Interaction p-values were reported as nominal and adjusted in the text with Bonferroni correction.

We performed four sensitivity analyses. i) extending the results to all SGLT2i; ii) excluding patients with CKD at baseline (eGFR <60 ml/min/1.73 m^2^ or UACR >30 mg/g); iii) excluding patients who initiated GLP-1RA in the comparator group (in these three sensitivity analyses, multiple imputation and PSM were repeated in the new datasets); iv) comparing the HR for categorical outcomes defined with or without a confirmatory eGFR value at least 90 days apart.

The conventional statistical significance threshold of 0.05 was used, without a hierarchical testing, except that secondary endpoints were analysed only in case significance on the primary endpoint was met. The analyses were run in R 4.2.2, using the MatchIt, mice, glmmTMB, stats, and survival packages.

### Role of funding source

The external funding source had no role in study design, data analysis, and collection, manuscript writing, or decision to publish.

## Results

### Patient disposition and characteristics

**Fig. 1:**
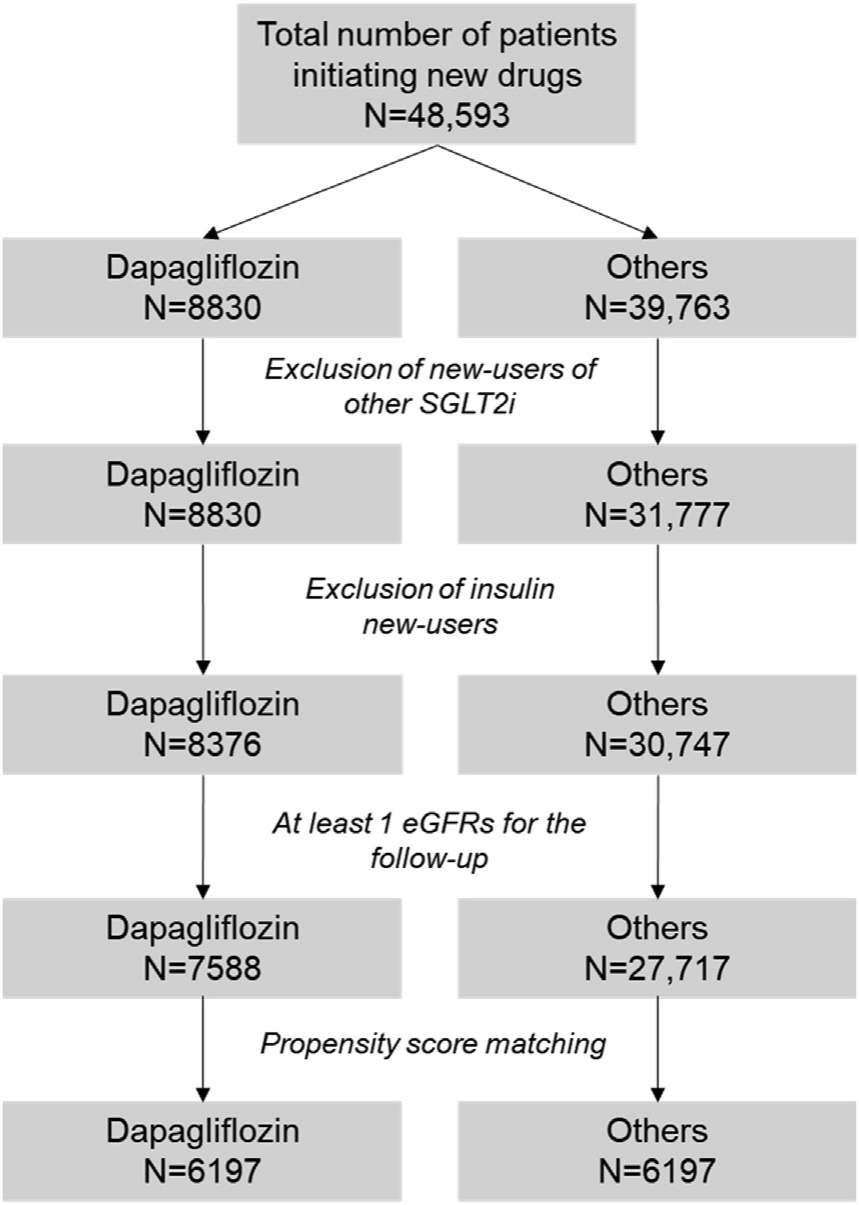
**Studyflowchart.**

Fifty centres provided data on 48,593 patients with T2D who initiated GLM of interest from 01/01/2015 to 30/09/2020 and were followed until 30/09/2021. Out of these, 8376 initiated dapagliflozin and 30,747 initiated other non-SGLT2i non-insulin GLM (Fig. 1). Before PSM, there were significant differences in most clinical characteristics between the two groups. Across the 10 imputed datasets, the matched cohorts were composed of an average of 6197 patients each (the number differed slightly across the various imputed datasets). After PSM, all characteristics were well balanced between groups, with all SMD <0.1 and often <0.01 (Table 1). Sixty-one percent of patients were males, the mean age was 61 and mean duration of diabetes was 10.5 years. Baseline BMI and HbA1c were 31.3 kg/m^2^ and 8.2% (66 mmol/mol), respectively. The prevalence of eGFR <60 ml/min/1.73 m^2^ was 6.4% and about 15% of patients had UACR >30 mg/g. The majority of patients (84%) were on metformin and about 30% were on insulin (basal and/or boluses). The prevalence of those using renin angiotensin system (RAS) blockers was 56%. The comparator GLM were DPP-4 inhibitors (40%), GLP-1 receptor agonists (22.3%), sulphonylureas (16.1%), pioglitazone (8%), metformin (5.8%), or acarbose (4%).

### Differences in the change of eGFR between groups

**Fig. 2:**
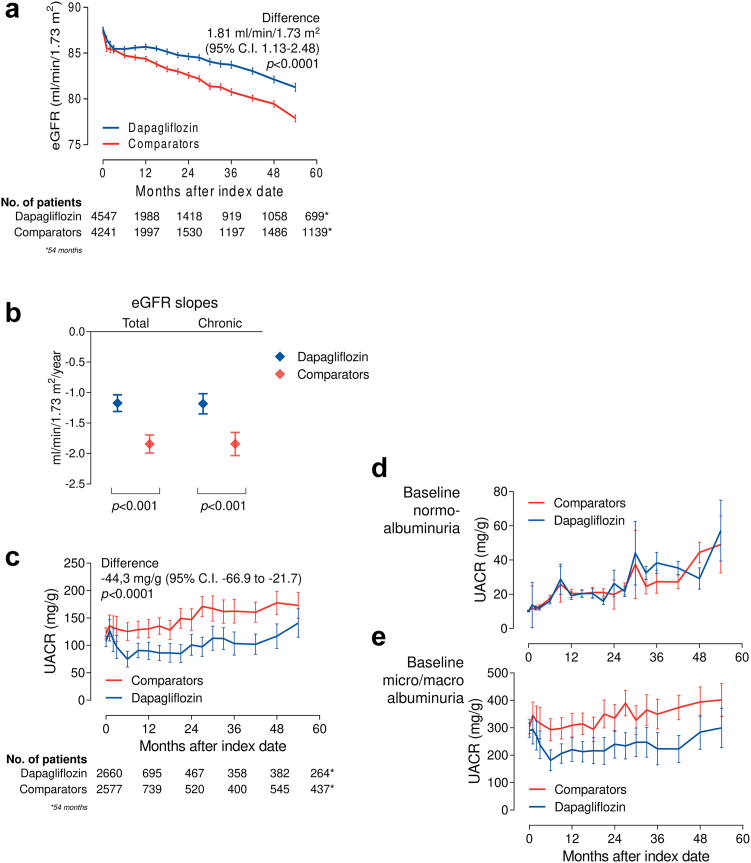
**Change in eGFR and albuminuria in the primary ITT analysis.** a) Change in eGFR over time in the two groups (primary outcome). The table at the bottom of the panel shows the number of patients contributing with data at each time point. This is the primary analysis performed, on average on a total of 6197 patients/group contributing with eGFR values, though not all patients had available baseline eGFR. Baseline eGFR was imputed only for PSM, but imputed data were not used for outcome evaluation. b) Total and chronic (6 months on) eGFR slopes in the two groups (bars indicate 95% C.I.). c) Adjusted change in albuminuria (urinary albumin excretion rate, UACR) over time in the two groups and in the split of the population by baseline normo- (d) or micro-macroalbuminuria (e). The table at the bottom of the panel shows the number of patients contributing with data at each time point. The observation period was cut at 54 months.

The primary endpoint was the change in eGFR from baseline to the end of observation. The mean follow-up duration was 30.4 months, with median (IQR) 28.4 (14.7–44.8). The observation was closed at 54 months because, at later time points, the residual population dropped below 10%. The median (IQR) number of eGFR values post-index date was 6 (3–10) per patient. From a baseline of 87.5 ml/min/1.73 m^2^, eGFR declined significantly less among new-users of dapagliflozin than among new-users of comparator drugs. The mean between-group difference was 1.81 ml/min/1.73 m^2^ (95% C.I. 1.13–2.48) in favour of the dapagliflozin group (p < 0.0001; Fig. 2a). This finding was confirmed with the paired analysis (Supplementary Figure S1). Supplementary Figure S2 shows that the eGFR curves of the two groups run parallel up to 4 years before the index date and separate thereafter. The acute dip in eGFR was similar between groups and the eGFR curves never crossed. eGFR stabilized at 3 months in the dapagliflozin group and remained higher than in the comparator group from 6 months on.

The total mean annualized eGFR slope, calculated from index date to last observation was −1.17 (95% C.I. −1.03 to −1.31) ml/min/1.73 m^2^/year in the dapagliflozin group and −1.84 (95% C.I. −1.70 to −1.99) ml/min/1.73 m^2^/year in the control group. The between-group difference was 0.67 (95% C.I. 0.47–0.88) ml/min/1.73 m^2^/year (p < 0.0001). The analysis of chronic slopes, calculated from 6 months after index date to the last observation, yielded a similar between-group difference was 0.66 ml/min/1.73 m^2^/year (p < 0.0001; Fig. 2b).

### Differences in the change of albuminuria between groups

This analysis was performed on the subset of patients with available data on UACR (n = 5237) and a median of 5 (IQR 3–9) values per patient. Albuminuria was measured in the morning void relative to urinary creatinine (88.6%) or in the daily urinary collection (11.4%). Though only 15% had baseline micro- or macro-albuminuria, the mean value was in the microalbuminuric range due to the highly skewed distribution. In 7 out of 10 imputed datasets, the prevalence of concomitant treatment with basal insulin was imbalanced between groups and needed to be adjusted for. UACR declined significantly in new-users of dapagliflozin during the first 6 months and remained lower than in new-users of comparators for the entire observation. The mean adjusted between group difference was −44.3 mg/g (95% C.I. −66.9 to −21.7; p < 0.0001; Fig. 2c). In patients with baseline normoalbuminuria, the change in UACR over time did not differ between the two groups, whereas the effect of dapagliflozin in the whole population was observed in patients with baseline micro- or macro-albuminuria. In addition, among patients with micro- or macro-albuminuria, the rate of albuminuria class improvement was significantly greater with dapagliflozin than with comparators (399 per 1000 person-years on dapagliflozin vs 315 per 1000 person year on comparators: HR 1.22; 95% C.I. 1.03–1.46; p = 0.030).

### Kidney function outcomes

**Fig. 3:**
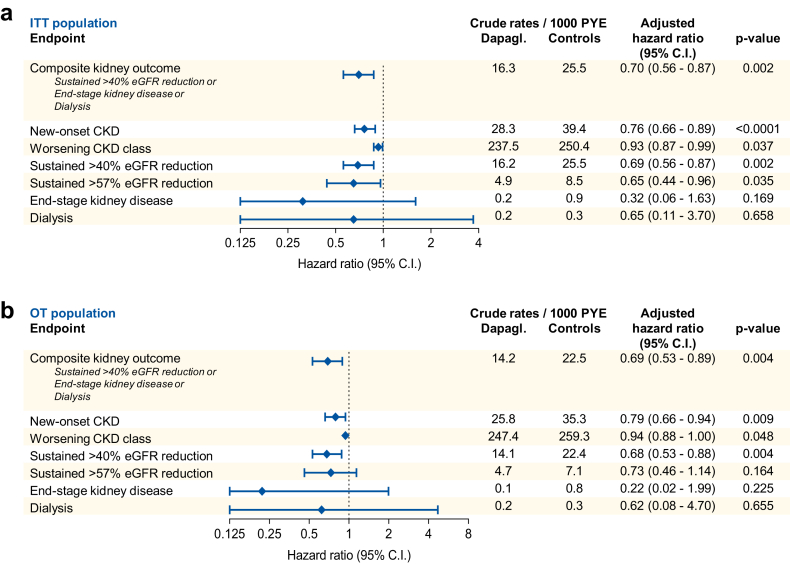
**Summary of results.** The forest plot show, for the intention-to-treat (ITT, a) and the on-treatment (OT, b) populations, the crude rates/1000 patient year events (PYE), the hazard ratio (HR) with 95% confidence intervals (C.I.) and the respective p-values for each categorical outcome.

**Fig. 4:**
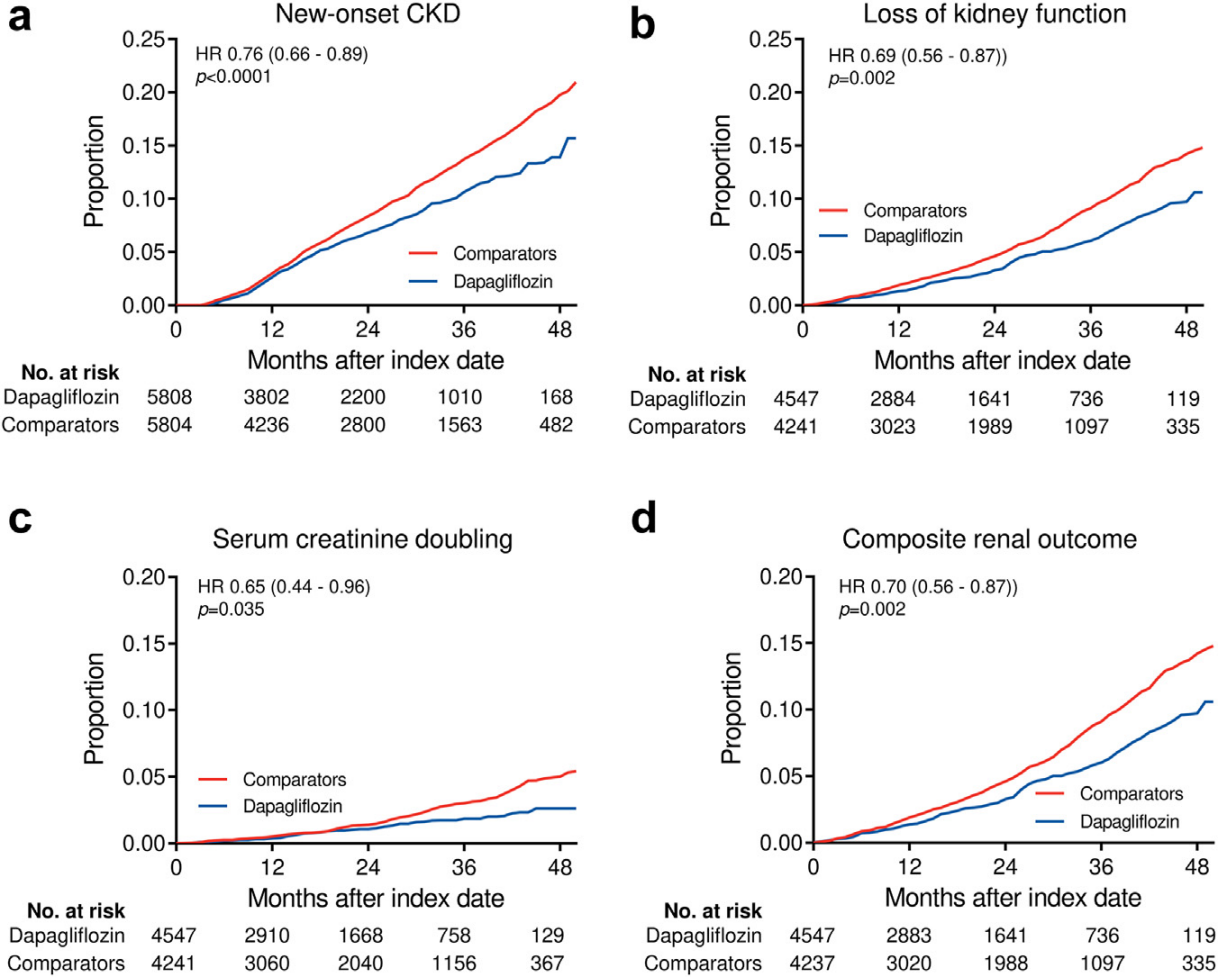
**Kaplan–Meier curves for selected outcomes.** Data from Cox proportional hazard models were used and the cumulative proportion of patients with an event is shown. Hazard ratios (HR) with 95% confidence intervals (C.I.) are presented. Number of patients at risk at each time points are also displayed.

We analysed a series of standard categorical outcomes reflecting loss of kidney function over time based on eGFR (Fig. 3a). New-users of dapagliflozin had a lower rate of confirmed new-onset CKD (HR 0.76; 95% C.I. 0.66–0.89; p < 0.0001), substantial loss of kidney function, defined as ≥40% or ≥57% reduction in eGFR, with respective HR of 0.69 (95% C.I. 0.56–0.87; p = 0.002) and 0.65 (95% C.I. 0.44–0.96; p = 0.035). The rate of a composite outcome of ≥40% loss of kidney function, end-stage kidney disease, or dialysis was also significantly lower in the dapagliflozin group (HR 0.70; 95% C.I. 0.56–0.87; p = 0.002). This finding was confirmed with the paired analysis (Supplementary Figure S1). Patients initiating dapagliflozin showed a protection against CKD class change (HR 0.93; 95% C.I. 0.87–0.99; p = 0.037). Kaplan–Meier survival curves for selected outcomes are shown in Fig. 4. A sensitivity analysis requiring a confirmatory eGFR at ≥90 days apart for all outcomes produced superimposable results in terms of HR, but with larger confidence intervals because the number of events was smaller (Supplementary Table S1).

### Intermediate endpoints

**Fig. 5:**
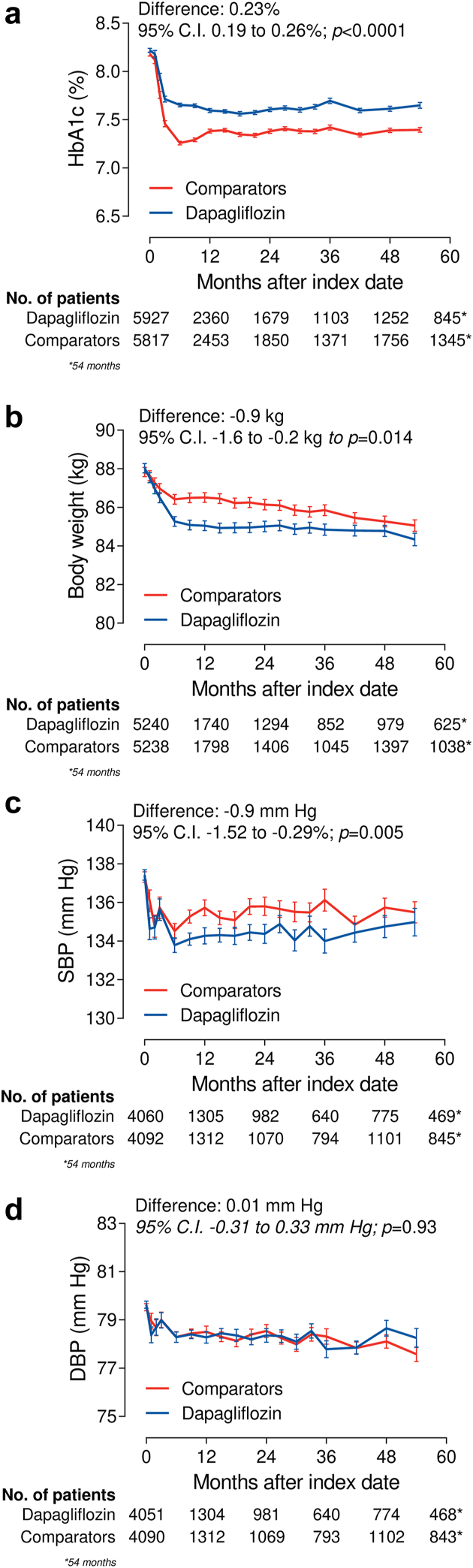
**Effects on intermediate endpoints.** The panels show the change over time in HbA1c (a), body weight (b), systolic blood pressure (SBP, c) and diastolic blood pressure (DBP, d) in the dapagliflozin and comparator groups. Mean differences between groups are reported on the top part. The table at the bottom of each panel shows the number of patients contributing with data at each time point. Note that the observation period was cut at 54 months.

HbA1c declined significantly more in the control group than in the dapagliflozin group, with a mean difference of 0.23% (Fig. 5a). Body weight declined significantly more in the dapagliflozin group than in the control group by 0.9 kg (Fig. 5b), as did systolic blood pressure (mean difference −0.9 mm Hg; Fig. 5c). The change in diastolic blood pressure was similar in the two groups (Fig. 5d). Patients who initiated dapagliflozin had a significantly greater rate of discontinuation of RAS blockers (39 vs 33 events/1000 patient-year; HR 1.28; 95% C.I. 1.08–1.52; p = 0.007) and thiazide diuretics (63 vs 50 events/1000 patient-year; HR 1.38; 95% C.I. 1.06–1.81; p = 0.020). The rate of discontinuation of loop diuretics was not significantly different (HR 1.42; 95% C.I. 0.93–2.18; p = 0.110).

### Subgroup and sensitivity analyses

The analyses were repeated in the on-treatment dataset, with patients censored at discontinuation of index drugs. Across the 10 imputed datasets, on average, 6110 patients per group were included and the mean observation time was 25.5 months. For the primary endpoint, eGFR declined significantly less in the dapagliflozin than in the comparator group, with a mean difference of 1.78 ml/min/1.73 m^2^ (Supplementary Figure S3a). UACR declined significantly with dapagliflozin and the adjusted mean difference between groups was −34.3 mg/g (Supplementary Figure S3b). Results on categorical endpoints based on eGFR were in line with the primary ITT analysis, although confidence intervals tended to be larger due to the shorter follow-up time (Fig. 3b).

Results were confirmed when the analyses were performed on 10,918 initiators of any SGLT2i (53% dapagliflozin, 39% empagliflozin, 8% canagliflozin) vs 10,918 matched initiators of comparators (Supplementary Table S3): the difference in the change in eGFR was 1.61 ml/min/1.73 m^2^ and the difference in the change in UACR was −34.5 mg/g (Supplementary Figure S4a and b). All categorical eGFR-based outcomes were in favour of SGLT2i, reaching statistical significance also for ESKD (Supplementary Figure S4c–e), and when confirmed by a second eGFR >90 days apart (Supplementary Table S1b).

When the analysis was repeated in the dataset of patients without CKD (4969/group; Supplementary Table S3), the eGFR over time was still significantly higher in the dapagliflozin than in the comparator group by 1.80 ml/min/1.73 m^2^ (Supplementary Figure S5a). As all patients were normoalbuminuric, consistently with the primary results, there was no difference in the change in albuminuria between the two groups (Supplementary Figure S5a). The rates of new-onset CKD (HR 0.75; 95% C.I. 0.63–0.88; p < 0.0001) and ≥40% loss of kidney function (HR 0.69; 95% C.I. 0.50–0.93; p = 0.02) were significantly lower in the dapagliflozin group (Supplementary Figure S5c–e).

The analysis run excluding GLP-1RA as comparators contained 5609 matched patients per group with an overall prevalence of CKD of 20% (Supplementary Table S4). In the dapagliflozin group, eGFR declined significantly less by 1.87 ml/min/1.73 m^2^ (Supplementary Figure S6a) and albuminuria was significantly lower by 43.6 mg/g (Supplementary Figure S4b). Rates of new-onset CKD (HR 0.76; 95% C.I. 0.64–0.91; p = 0.005), ≥40% loss of kidney function (HR 0.68; 95% C.I. 0.54–0.85; p = 0.001), creatinine doubling (HR 0.61; 95% C.I. 0.41–0.93; p = 0.02), and the composite kidney outcome (HR 0.68; 95% C.I. 0.54–0.85; p = 0.001) were all significantly lower in the dapagliflozin vs comparator group (Supplementary Figure S6c–e).

**Fig. 6:**
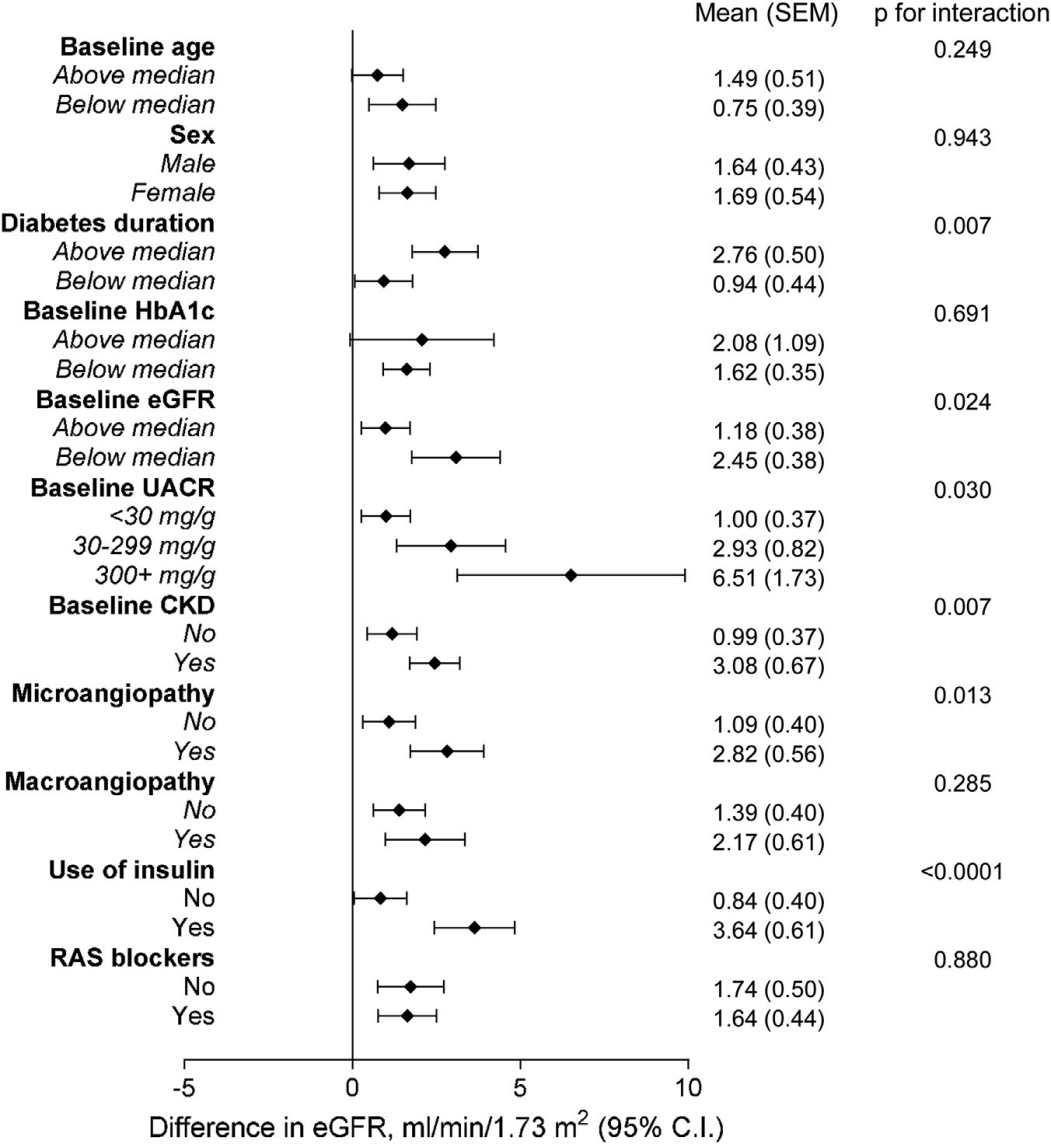
**Subgroup analysis.** For the primary endpoint (change in eGFR), the analysis was repeated in subgroups of patients based on key clinical characteristics at baseline. The mean change in eGFR is reported for each strata and the p-values for interaction are also displayed.

For the primary endpoint, we performed a subgroup analysis, with patients stratified based on pre-specified baseline characteristics. We calculated the mean difference in the change of eGFR in each stratum, along with the respective p-value for interaction. All point estimates were in favour of dapagliflozin and most effects in the various strata were statistically significant. According to nominal p-values for interaction, we found better preservation of eGFR over time with dapagliflozin vs comparators in patients with longer diabetes duration, below-median baseline eGFR, CKD, UACR>30 mg/g, microangiopathy, or using insulin. After Bonferroni correction, the p-value for interaction of the stratification by use of insulin remained significant (Fig. 6).

## Discussion

In a population of outpatients with T2D and a low prevalence of CKD, we found that new-users of dapagliflozin, as compared to new-users of other glucose lowering medications, were protected from the decline in kidney function, the rise in albuminuria, and the occurrence of a composite adverse kidney outcome. Remarkably, dapagliflozin preserved kidney function also in patients without CKD at baseline. The findings were statistically robust in all sensitivity analyses and clinically meaningful with respect to the degree of protection conferred by dapagliflozin. In the primary analysis, over a mean of 2.5 years, those initiating dapagliflozin had higher eGFR values by about 1.81 ml/min/1.73 m^2^, less negative chronic eGFR slope by about 0.7 ml/min/1.73 m^2^/year, lower albuminuria by 44 mg/g, and a relative 30% lower rate of substantial loss of kidney function, ESKD or dialysis. According to prior studies, such difference in eGFR slopes provides a very high positive predictive value for a nonzero benefit on ESKD,^28^ which is the hardest outcome in terms of kidney disease prevention. Notably, a significant protection against ESKD emerged when the analysis was extended to all SGLT2i. Based on absolute rates of events, we estimate that 22 patients needed to be treated with dapagliflozin for 5 years to prevent one composite kidney endpoint (18 for new-onset CKD), which can be considered a remarkable benefit in the field of kidney disease prevention.^29^ This is particularly true considering that baseline eGFR was 87 ml/min/1.73 m^2^, only 6% of patients had baseline CKD stage III or higher and only 15% had micro- or macro-albuminuria. In fact, the population under investigation in our study was composed mostly of patients in so-called “primary renal prevention”. The most likely reason for the low prevalence of CKD in this population is the restriction to initiation of SGLT2i in patients with reduced eGFR, which was ongoing for most of the period of data collection. To our knowledge, this is the first demonstration that dapagliflozin protects patients with normal eGFR and albuminuria from the loss of kidney function.

Our results resemble those of the DECLARE-TIMI 58^30^ wherein dapagliflozin significantly reduced the rate of adverse kidney outcomes, across all KDIGO risk categories. Notably, our study population had baseline age, sex, BMI, diabetes duration and eGFR similar to those in the trial, though DECLARE-TIMI 58 included about 40% of patients who had experienced a cardiovascular event, as opposed to 12% in our study.

Our findings are also in line with an increasing wealth of real-world evidence on the kidney protective effects of SGLT2i.^15^ Earlier studies used administrative data to compare hard kidney outcomes among new-users of SGLT2i or other medications,^31^^,^^32^ whereas more recent studies reported data on eGFR trajectories or slopes.^33^, ^34^, ^35^ Among the latter group of studies, CVD-Real 3 was the largest one, as it included about 76,000 new-users of SGLT2i or comparators (including insulin) from a multinational cohort. The primary endpoint was the eGFR slope and data on albuminuria were not presented. To calculate the eGFR slope, the CVD-Real 3 protocol imposed specific constrains in the numbers and timings of pre- and post-index date eGFR values. Initiating treatment with SGLT2i was associated with an acute drop in eGFR followed by an increase and subsequent stabilization, while the control group's eGFR continued to decline.^14^

Recently, upon a critical review of the literature, we identified several unmet issues in these real-world studies, that we planned to address in DARWIN-Renal. To reduce confounding by indication, we applied gold standard methodologies of comparative effectiveness research. PSM was performed on several clinical and laboratory variables and yielded an optimal balance between groups. Given that matched patients had the same post-hoc probability of receiving either treatment (PS), this approach makes observational research closer to randomized trials, where all patients have 50% probability of receiving each treatment. To allow estimation of PS for patients with missing data, instead of matching on the missing category, we performed all analyses in 10 imputed datasets and then pooled results. Furthermore, to limit residual confounding, we excluded patients who initiated insulin because, at means of covariates, insulin initiation is always a proxy of disease severity that, in observational research, drives poor outcomes.^36^, ^37^, ^38^ As noted before,^15^ pooling results obtained in different databases is problematic due to heterogeneity in duration of observation and sampling rates. To overcome this limitation, we collected data from the same type of electronic medical records of patients followed under the same model of specialist care, which is highly homogeneous across the Country. We ruled out a significant time-leg bias^39^ by the new-user design, wherein all patients were observed since initiation of new treatments in the same period, after commercialization of dapagliflozin in Italy. In addition, matching on several clinical variables that represent disease severity (age, diabetes duration, prior eGFR slope, background medications, and complications) ensured that patients in the two cohorts were in the same disease stage.^40^ We kept conditioning on the future to a minimum. Contrary to what was done in CVD-Real 3 (requiring specific timings and numbers of eGFR values), we imposed that only one eGFR value post-index date was available (otherwise the primary outcome could not be analysed) without constrains on the timing of updated eGFR values. Although patients needed to be alive to collect endpoint data, any immortal time would apply to the same extent in both groups. Analysing eGFR linear slope can be problematic, especially when non-linear trends are present (e.g., after initiation of SGLT2 inhibitors), which can lead to biologically implausible positive slopes.^14^ To avoid this artefact, we used the MMRM approach, which makes no assumption on the shape of eGFR curves. Although computationally more intensive, this model yields the mean difference over time between groups, including periods of non-linear trends. To compare with the existing literature and with the approved surrogate endpoints,^28^ we also calculated eGFR slopes but, in order to exclude the acute eGFR dip in new-users of SGLT2 inhibitors, we pre-specified the analysis of a chronic slope, starting 6 months after index date for each participant. This analysis provided a slope of approximately −2 ml/min/1.73 m^2^/year in the comparator group, which is consistent with the expected eGFR decline in patients with T2D and normal baseline kidney function.^41^ The between-group slope difference was in line with what expected from trial results in a similar population.^42^ Finally, for these estimates to be reliable, an observation longer than 2 years is usually required,^28^ and our study had twice as much the median follow-up of the CVD-Real 3 study,^14^ i.e., 30 vs 15 months.

Another strength of our study is the analysis of the effect of dapagliflozin on albuminuria. Although we already found that dapagliflozin reduced albuminuria after 6 months of treatment in a much smaller population,^19^ we now report the long-term persistence of such effect in a larger population. The difference in albuminuria between new-users of dapagliflozin or comparators developed within the first 6 months and maintained for the entire observation up to 54 months. Dapagliflozin increased the probability of regression from micro-/macro-albuminuria similarly to what observed in DECLARE-TIMI 58,^43^ but the improvement in albuminuria was observed only among patients with UACR >30 mg/g at baseline.

Thanks to the availability of intermediate endpoints, we can underline that the striking superiority of dapagliflozin vs comparators on kidney outcomes was achieved with modest differences in blood pressure and body weight and with slightly higher HbA1c values throughout observation. This difference in HbA1c is probably due to reimbursement restrictions that applied during the study period to the drop-in drugs that could not be associated with SGLT2i (DPP-4 inhibitors and GLP-1RA). Nonetheless, these finding suggests that renal protection by dapagliflozin may be due to a direct effect rather than to the improvement of risk factors for kidney function decline. Of note, we found a similar acute dip in eGFR in the two groups. This may be due to the rapid decline in HbA1c within the first 3–6 months in the comparator group, which is predicted to reduce eGFR transiently.^44^^,^^45^ In the primary analysis, 22.3% of patients in the comparator group were new-users of GLP-1RA, which could lead to an underestimation of the true effects of dapagliflozin, because GLP-1RA can exert some degree of renal protection.^46^ Excluding GLP-1RA initiators from the comparator group did not modify the findings and allowed to achieve statistical significance also for the protection against the doubling of serum creatinine.

Limitations of our study are mainly intrinsic to its observational design. First, despite successful matching on several clinical-laboratory variables, we cannot exclude residual confounding due to measurement error and unmeasured factors. For example, patients in the dapagliflozin group had marginally higher baseline eGFR values and marginally lower pre-index date eGFR slopes. Regarding unmeasured features, we had no data on socio-economic variables. Though they were unlikely to affect the exposure-outcome association because all patients were followed under the same specialist care system under full public healthcare coverage, there is an increasing awareness that deprivation statuses can worsen CKD outcomes.^47^ Second, the density of updated values for eGFR and UACR in routine care is typically higher in patients with prevalent kidney disease, whereas patients with normal kidney function are less likely to have their eGFR and UACR re-checked at short intervals. Without constrains on the availability of post-index date values, this pattern may favour the null hypothesis, a conservative approach that reduced the risk of false positive findings. Nonetheless, imposing a confirmatory eGFR value >90 days apart for categorical outcomes did not change HRs meaningfully. Third, although we confirmed results in the OT population based on prescriptions, we had no data on treatment adherence and on the reasons for drug discontinuation or side effects. We also acknowledge the built-in selection bias in HRs^48^ and the small number of ESKD and dialysis events in the primary analysis, generating unrealistically large confidence intervals (sparse data bias^49^). Finally, the data source contained no information on cardiovascular events and death, which can compete with kidney outcomes. Under the assumption that dapagliflozin can reduce cardiovascular events and mortality,^50^ this limitation also favours the null hypothesis and does not increase the risk of false positive findings.

In summary, our study, designed to overcome some issues with prior observational research, supports the strong protective effects of dapagliflozin against the loss of kidney function under routine care, even in patients without baseline CKD. Based on real-world evidence, it is expected that broadening the population of patients with T2D who are receiving SGLT2i will drive a change in the epidemiology of ESKD in the next decades.

## Contributors

GPF conceptualised the study, interpreted the analyses, visualised the results, searched the literature, and wrote the manuscript. EL conceptualised the study, performed and interpreted the analyses, visualised the results, developed the methodological pipeline, searched the literature, and contributed to write the manuscript. MLM made substantial contributions to the acquisition and elaboration of research data. AA, SDP, and AS conceptualised the study, coordinated and supervised data collection, acquired funding for the analysis, and critically reviewed the manuscript for important intellectual content. All authors contributed intellectually to this study and critically revised the scientific content of the manuscript. All authors had access to all the data of the study, approved the final manuscript as submitted, agreed to be accountable for all aspects of the work, and had final responsibility for the decision to submit for publication. GPF and EL are the guarantors of this work and, as such, had full access to all the data in the study, verified the data, and take responsibility for the integrity of the data and the accuracy of the data analysis.

## Data sharing statement

Restrictions apply to the availability of crude data used for this study. Aggregated data are available upon reasonable request via email to the corresponding author.

## Declaration of interests

GPF received fees for lectures, consultancy, or advisory board from Abbott, AstraZeneca, Boehringer, Lilly, MSD, Mundipharma, Novo Nordisk, Sanofi, Servier, Takeda. MLM received lecture or consultancy fees from AstraZeneca, Lilly, MSD, Mylan, Novo Nordisk, SlaPharma, and Servier. SDP consulted for Applied Therapeutics, AstraZeneca, Boehringer Ingelheim, Eli Lilly, MSD, Novartis, Novo Nordisk, and Sanofi, and received funding for these consulting services; received grant support from AstraZeneca and Boehringer Ingelheim; and received speaker fees from AstraZeneca, Boehringer Ingelheim, Eli Lilly, MSD, Novartis, Novo Nordisk, and Sanofi. AA received research grants, lecture, or advisory board fees from Merck Sharp & Dome, AstraZeneca, Novartis, Boeringher-Ingelheim, Sanofi, Mediolanum, Janssen, Novo Nordisk, Lilly, Servier, and Takeda. AS served on the advisory board of Novo Nordisk, Sankyo, and Sanofi and received grant support from Sankyo and speaker fees from Astra Zeneca, Bayer, Lilly, Novo Nordisk, and Sanofi. EL has nothing to disclose.
