# Supplementary material for: Long-term benefits of dapagliflozin on renal outcomes of type 2 diabetes under routine care: a comparative effectiveness study on propensity score matched cohorts at low renal risk
Source: Lancet Reg Health Eur. 2024 Feb 1;38:100847. doi: 10.1016/j.lanepe.2024.100847 (PMC10847023; doi:10.1016/j.lanepe.2024.100847)

Online Data Supplement

Table of contents

Content	*Page*
DARWIN-Renal study Investigators	2
Table S1. Sensitivity analysis with confirmatory eGFR at ≥90 days	4
Table S2. Clinical characteristics of initiators of SGLT2i or comparators	5
Table S3. Clinical characteristics of cohorts of patients without CKD	7
Table S4. Clinical characteristics of cohorts of patients in the analysis excluding GLP-1RA as comparators	9
Figure S1. Comparison of the paired and unpaired analytical approach	11
Figure S2. Plot of pre- and post-index date eGFR values	12
Figure S3. Results of the on-treatment analyses	13
Figure S4. Main results of the comparison between initiators of SGLT2 inhibitors and initiators of comparators	14
Figure S5. Main results of the cohort of patients without baseline CKD	15
Figure S6. Main results of the cohort of patients without GLP-1RA new-users in the comparator group	16

DARWIN-Renal study Investigators

ABRUZZO. Mariella Baldassarre, Agostino Consoli (Endocrinology, Diabetes and Metabolism, University Chieti-Pescara). Sara Morganet, Antonella Zugaro, Marco Giorgio Baroni (Diabetes and Andrology Unit, San Salvatore Hospital, University of L'Aquila).

CALABRIA. Francesco Andreozzi (Department of Medical and Surgical Sciences, University Magna Graecia of Catanzaro, Catanzaro).

CAMPANIA. Adriano Gatti (Diabetology Service, ASL Napoli1). Stefano De Riu (Diabetology Unit ASL Napol1 Centro). Andrea Del Buono (Diabetology Unit, ASL Caserta).

EMILIA ROMAGNA. Raffaella Aldigeri, Riccardo Bonadonna (Division of Endocrinology and Metabolic Diseases, Azienda Ospedaliera-Universitaria di Parma,, Department of Medicine and Surgery, University of Parma), Alessandra Dei Cas (Division of Nutritional and Metabolic Sciences, Azienda Ospedaliero-Universitaria di Parma, University of Parma), Angela Vazzana, Monica Antonini, Valentina Moretti (Division of Endocrinology and Metabolic Diseases, Azienda Ospedaliera-Universitaria di Parma).

FRIULI VENEZIA GIULIA. Patrizia Li Volsi (Section of Endocrinology and Metabolism, Azienda Sanitaria Friuli Occidentale). Miranda Cesare, Giorgio Zanette (Section of Endocrinology and Metabolism, Pordenone Hospital, Azienda Sanitaria Friuli Orientale).

LAZIO. Silvia Carletti, Paola D'Angelo (Diabetology Unit, Sandro Pertini Hospital, ASL Roma2). Gaetano Leto, Frida Leonetti (Diabetology Unit Latina, Department of Medical-Surgical Sciences and Biotechnologies, Sapienza University, Rome, Italy). Luca D'Onofrio, Ernesto Maddaloni, Raffaella Buzzetti (Diabetology Unit, University of Rome "La Sapienza"). Simona Frontoni (Unit of Endocrinology, Diabetes and Metabolism, S. Giovanni Calibita Fatebenefratelli Hospital, Department of Systems Medicine, University of Rome Tor Vergata, Rome, Italy). Giselle Cavallo (Diabetology Unit, Department of Experimental Medicine, Sapienza University, Rome, Italy). Susanna Morano, Tiziana Filardi (University of Rome "La Sapienza", Dept. of Experimental Medicine, Unit of Diabetes and Complications, Clinica Medica V, Azienda Policlinico Umberto I, Roma). Umberto Capece, Andrea Giaccari (Endocrinology and Diabetology, IRCCS Agostino Gemelli University Hospital Foundation, Department of Translational Medicine and Surgery, Catholic University of the Sacred Heart, Rome).

LOMBARDIA. Antonio C. Bossi (Unit of Diabetology, Humanitas Gavazzeni Institute, Bergamo). Giancarla Meregalli (Unit of Diabetology and Metabolic Diseases, Azienda Socio-Sanitaria Territoriale Bergamo Ovest, Treviglio, Bergamo). Fabrizio Querci (Diabetology Unit, Alzano Lombardo Hospital). Alessia Gaglio, Veronica Resi, Emanuela Orsi (Diabetes Unit, Foundation IRCCS Cà Granda Ospedale Maggiore Policlinico, Milan). Stefano Fazion (Diabetology and Metabolic Disease, ASST Mantova). Ivano G. Franzetti (Endocrinology and Diabetology Unit ASST Valle Olona). Cesare Berra (Diabetology and Endocrinology Unit, IRCCS Multimedica, Milan).

MARCHE. Silvia Manfrini (Diabetology Unit, Senigallia). Gabriella Garrapa, Giulio Lucarelli, Lara Riccialdelli (Diabetology Unit, Fano). Elena Tortato (Diabetology and Metabolic Disease, INRCA Ancona).

PIEMONTE. Marco Zavattaro, Gianluca Aimaretti (Division of Endocrinology, Maggiore della Carità Hospital, Department of Translational Medicine, University of Piemonte Orientale, Novara). Franco Cavalot (Diabetes and Metabolic Diseases Unit, San Luigi Gonzaga University Hospital, Turin). Guglielmo Beccuti, Fabio Broglio (Unit of Diabetology and Metabolism, Department of Medical Sciences, University of Turin, 10123 Torino, Italy.).

TRENTINO-ALTO ADIGE. Bruno Fattor (Diabetology Service, Bolzano Hospital).

PUGLIA. Giuliana Cazzetta (Diabetology Unit Tricase (Lecce)). Olga Lamacchia (Department of Medical and Surgical Sciences, University of Foggia). Anna Rauseo, Salvatore De Cosmo (Fondazione IRCCS Casa Sollievo della Sofferenza, San Giovanni Rotondo, Italy).

SARDEGNA. Rosella Cau, Mariangela Ghiani (UO diabetologia Quartu SE, ASL 8 Cagliari).

SICILIA. Antonino Di Benedetto (Diabetology Unit, University Hospital G. Martino, Messina). Antonino Di Pino, Salvatore Piro, Francesco Purrello (Internal Medicine, Garibaldi Nesima Hospital, Department of Clinical and Experimental Medicine, University of Catania). Lucia Frittitta, Agostino Milluzzo (Center for Diabetes and Obesity, Garibaldi Nesima Hospital, Department of Clinical and Experimental Medicine, University of Catania). Giuseppina Russo (Metabolic Disease and Internal Medicine, University Hospital of Messina).

TOSCANA. Anna Solini (Department of Surgical, Medical, Molecular and Critical Area Pathology, University of Pisa). Monia Garofolo, Giuseppe Penno, Stefano Del Prato (Department of Clinical and Experimental Medicine, University of Pisa, Pisa). Roberto Anichini (Diabetes Unit, Area Pistoiese USL Toscana Centro).

VENETO. Gian Paolo Fadini, Angelo Avogaro (Department of Medicine, University of Padova). Lucia Gottardo (Unit of Hypertension and Endocrine-Metabolic-Angiologic Disease, AULSS3 Venice). Mauro Rigato, Agostino Paccagnella (Diabetology Units, Conegliano and Treviso). Marco Strazzabosco (Endocrine, Metabolic and Nutrition Disease, Vicenza Hospital). Massimo Cigolini, Enzo Bonora (Division of Endocrinology, Diabetes and Metabolic Diseases, Department of Medicine, University of Verona).

Table S1. Sensitivity analysis with confirmatory eGFR at ≥90 days. Results are reported as hazard ratios (95% confidence intervals) for the difference between dapagliflozin (A) or all SGLT2 inhibitors (B) versus comparators (HR<1.0 indicates lower hazard in the dapagliflozin group). Note that new-onset CKD and end-stage kidney disease required a confirmation eGFR in the primary analysis and is therefore not included here.

A) Dapagliflozin versus comparators

Outcome	Without confirmatory eGFR	With confirmatory eGFR
>40% eGFR reduction	0.69 (0.56 - 0.87)	0.67 (0.43 - 1.04)
>57% eGFR reduction	0.65 (0.44 - 0.96)	0.58 (0.26 - 1.29)
Composite renal outcome	0.70 (0.56 - 0.87)	0.68 (0.44 - 1.06)

B) SGLT2 inhibitors versus comparators

Outcome	Without confirmatory eGFR	With confirmatory eGFR
>40% eGFR reduction	0.72 (0.61 - 0.84)	0.68 (0.49 - 0.93)
>57% eGFR reduction	0.58 (0.43 - 0.78)	0.50 (0.26 - 0.96)
Composite renal outcome	0.71 (0.60 - 0.84)	0.67 (0.49 - 0.92)

Table S2. Clinical characteristics of initiators of SGLT2i or comparators. Data are presented for patients who initiated SGLT2 inhibitors (SGLT2i) or comparators before and after propensity score matching. Continuous variables are presented as mean (standard deviation) and median (IQR). Categorical variables are presented as number (percentage).

	Before PSM			After PSM		
	**SGLT2i**	**Comparators**	**SMD**	**SGLT2i**	**Comparators**	**SMD**
**Number**	14495	27893		10918	10918	
**Demographics**						
Sex male, n (%)	9025 (62.3)	16907 (60.6)	0.03	6922 (63.4)	6903 (63.2)	<0.01
Age, years	61.4 (8.9)	63.6 (8.8)	0.25	61.3 (9.0)	61.5 (9.5)	0.02
Diabetes duration, years	11.8 (8.6)	10.6 (7.9)	0.14	10.2 (7.9)	10.2 (8.1)	<0.01
**Anthropometrics**						
Weight, kg	88.7 (18.1)	83.9 (17.9)	0.27	87.7 (17.8)	87.5 (19.0)	0.01
Height, cm	167.3 (9.7)	166.6 (9.7)	0.08	167.5 (9.7)	167.4 (9.7)	<0.01
Body mass index, kg/m^2	31.7 (5.8)	30.2 (5.8)	0.25	31.2 (5.7)	31.2 (6.1)	<0.01
Waist, cm	109.0 (13.4)	105.5 (13.4)	0.26	108.0 (13.3)	107.7 (13.8)	0.02
**Risk factors and laboratory**						
Systolic blood pressure, mm Hg	137.6 (18.9)	136.4 (18.4)	0.07	136.9 (18.6)	136.9 (18.4)	<0.01
Diastolic blood pressure, mm Hg	79.1 (10.1)	78.2 (9.9)	0.09	79.3 (10.0)	79.3 (10.1)	<0.01
Fasting plasma glucose, mg/dl	173.0 (58.3)	159.6 (48.0)	0.26	167.1 (54.0)	166.3 (52.5)	0.02
HbA1c, %	8.4 (1.5)	7.8 (1.2)	0.39	8.1 (1.4)	8.1 (1.4)	0.02
Total cholesterol, mg/dl	173.4 (44.2)	172.8 (43.1)	0.01	173.1 (43.4)	173.0 (43.9)	<0.01
HDL cholesterol, mg/dl	46.6 (14.4)	48.7 (15.4)	0.14	46.8 (14.3)	47.0 (14.6)	0.01
LDL cholesterol, mg/dl	94.6 (36.8)	94.4 (35.9)	<0.01	94.5 (36.2)	94.9 (36.6)	0.01
Triglycerides, mg/dl	168.3 (123.4)	154.9 (111.5)	0.12	166.4 (121.4)	163.3 (126.0)	0.03
eGFR, ml/min/1.73 m^2	86.9 (16.6)	80.7 (21.0)	0.32	87.1 (16.7)	87.0 (17.8)	<0.01
Albumin excretion rate, mg/g	65.5 (469.9)	65.7 (407.3)	<0.01	59.6 (506.4)	57.1 (259.7)	<0.01
eGFR slope, ml/min/1.73 m^2/year	-0.7 (2.3)	-1.1 (2.3)	0.16	-0.7 (2.2)	-0.8 (2.2)	0.03
**Complications**						
Chronic kidney disease, n (%)	3031 (20.9)	7789 (27.9)	0.16	2208 (20.2)	2191 (20.0)	<0.01
eGFR <60 ml/min/1.73 m^2	886 (6.1)	4666 (16.7)	0.32	725 (6.6)	682 (6.2)	0.02
UACR >30 mg/g, n (%)	2364 (16.3)	4208 (15.1)	0.03	1657 (15.2)	1682 (15.4)	<0.01
Diabetic retinopathy, n (%)	2759 (19.0)	3757 (13.5)	0.15	1518 (13.9)	1463 (13.4)	0.01
Diabetic macular edema, n (%)	398 (2.7)	509 (1.8)	0.06	205 (1.9)	198 (1.8)	<0.01
Stroke / TIA, n (%)	216 (1.5)	442 (1.6)	<0.01	142 (1.3)	156 (1.4)	0.01
Carotid atherosclerosis, n (%)	2883 (19.9)	5926 (21.2)	0.03	2011 (18.4)	2073 (19.0)	0.01
Ischemic heart disease, n (%)	2115 (14.6)	3039 (10.9)	0.11	1462 (13.4)	1318 (12.1)	0.04
Left ventricular hypertrophy, n (%)	1124 (7.8)	2227 (8.0)	<0.01	797 (7.3)	764 (7.0)	0.01
Heart failure, n (%)	487 (3.4)	699 (2.5)	0.05	343 (3.1)	290 (2.7)	0.03
Any site revascolarization, n (%)	1493 (10.3)	2155 (7.7)	0.09	1048 (9.6)	934 (8.6)	0.04
Microvascular complications, n (%)	5547 (38.3)	11186 (40.1)	0.04	3689 (33.8)	3611 (33.1)	0.02
Macrovascular complications, n (%)	5163 (35.6)	9456 (33.9)	0.04	3650 (33.4)	3482 (31.9)	0.03
Established CVD, n (%)	2521 (17.4)	3816 (13.7)	0.10	1740 (15.9)	1592 (14.6)	0.04
**Glucose lowering medications**						
Metformin, n (%)	11523 (79.5)	21045 (75.4)	0.10	9234 (84.6)	9187 (84.1)	0.01
Sulphonylurea / repaglinide, n (%)	905 (6.2)	5401 (19.4)	0.37	899 (8.2)	898 (8.2)	<0.01

DPP-4 inhibitors, n (%)	174 (1.2)	3130 (11.2)	0.38	173 (1.6)	183 (1.7)	<0.01
GLP-1 receptor agonists, n (%)	223 (1.5)	1011 (3.6)	0.12	218 (2.0)	232 (2.1)	<0.01
Pioglitazone, n (%)	237 (1.6)	1234 (4.4)	0.15	230 (2.1)	271 (2.5)	0.03
Acarbose, n (%)	88 (0.6)	225 (0.8)	0.02	72 (0.7)	78 (0.7)	<0.01
Bolus insulin, n (%)	4255 (29.4)	720 (2.6)	0.91	726 (6.6)	699 (6.4)	0.01
Basal insulin, n (%)	6330 (43.7)	4544 (16.3)	0.66	2870 (26.3)	2665 (24.4)	0.04
**Other medications**						
Statins, n (%)	8321 (57.4)	15069 (54.0)	0.07	6077 (55.7)	5972 (54.7)	0.02
Anti-platelet agents, n (%)	5921 (40.8)	10515 (37.7)	0.06	4158 (38.1)	4036 (37.0)	0.02
RAS blockers, n (%)	8597 (59.3)	15837 (56.8)	0.05	6329 (58.0)	6276 (57.5)	<0.01
Beta blockers, n (%)	4385 (30.3)	7500 (26.9)	0.07	3191 (29.2)	3102 (28.4)	0.02
Calcium channel inhibitors, n (%)	3088 (21.3)	6015 (21.6)	<0.01	2255 (20.7)	2266 (20.8)	<0.01
Diuretics, n (%)	4212 (29.1)	8637 (31.0)	0.04	3058 (28.0)	3037 (27.8)	<0.01
Anticoagulants, n (%)	355 (2.4)	860 (3.1)	0.04	261 (2.4)	251 (2.3)	<0.01

Microvascular complications included retinopathy, neuropathy, and nephropathy. Macrovascular complications included evidence of atherosclerosis in any arterial site. Established cardiovascular disease (CVD) was defined as myocardial infarction, stroke or transient ischemic attack (TIA), or prior arterial revascularization. RAS, renin angiotensin system. SMD, standardized mean difference.

Table S3. Clinical characteristics of cohorts of patients without CKD. Data are presented for patients who initiated dapagliflozin or comparators before and after propensity score matching. Continuous variables are presented as mean (standard deviation) and median (IQR). Categorical variables are presented as number (percentage).

	Before PSM			After PSM		
	**Dapagliflozin**	**Comparators**	**SMD**	**Dapagliflozin**	**Comparators**	**SMD**
**Number**	6053	19967		4969	4969	
**Demographics**						
Sex male, n (%)	3579 (59.1)	11759 (58.9)	<0.01	2991 (60.2)	2991 (60.2)	<0.01
Age, years	60.5 (8.9)	62.7 (9.0)	0.24	60.5 (9.0)	60.5 (9.8)	<0.01
Diabetes duration, years	11.3 (8.2)	10.1 (7.7)	0.15	10.3 (7.7)	10.2 (8.0)	0.01
**Anthropometrics**						
Weight, kg	88.0 (18.3)	83.4 (18.0)	0.26	87.2 (17.9)	87.1 (19.3)	<0.01
Height, cm	167.1 (9.8)	166.5 (9.7)	0.06	167.3 (9.7)	167.3 (9.8)	<0.01
Body mass index, kg/m^2	31.5 (5.8)	30.0 (5.8)	0.25	31.1 (5.6)	31.1 (6.2)	<0.01
Waist, cm	108.7 (13.3)	104.9 (13.4)	0.29	107.8 (13.1)	107.4 (14.2)	0.02
**Risk factors and laboratory**						
Systolic blood pressure, mm Hg	136.8 (18.8)	135.8 (18.2)	0.06	136.4 (18.8)	136.1 (18.0)	0.02
Diastolic blood pressure, mm Hg	79.2 (10.2)	78.3 (9.7)	0.10	79.5 (10.2)	79.4 (9.7)	<0.01
Fasting plasma glucose, mg/dl	172.5 (56.0)	160.6 (47.4)	0.24	168.1 (53.1)	167.0 (52.9)	0.02
HbA1c, %	8.3 (1.5)	7.9 (1.2)	0.37	8.2 (1.4)	8.1 (1.4)	0.02
Total cholesterol, mg/dl	175.4 (44.8)	173.9 (43.7)	0.03	175.0 (44.1)	175.5 (44.9)	0.01
HDL cholesterol, mg/dl	46.9 (14.2)	49.2 (14.8)	0.16	47.2 (14.2)	47.6 (14.2)	0.03
LDL cholesterol, mg/dl	96.8 (38.2)	95.8 (37.6)	0.03	96.4 (37.6)	97.0 (37.8)	0.02
Triglycerides, mg/dl	164.6 (129.7)	149.8 (104.8)	0.13	163.6 (131.7)	160.3 (131.2)	0.03
eGFR (ml/min/1.73 m^2)	89.7 (14.2)	88.3 (14.0)	0.10	89.9 (14.2)	89.7 (14.6)	0.01
Albumin excretion rate, mg/g	10.0 (7.1)	10.2 (7.2)	0.02	9.9 (7.1)	10.0 (7.0)	<0.01
eGFR slope (ml/min/1.73 m^2/year)	-0.4 (1.9)	-0.5 (2.0)	0.01	-0.4 (2.0)	-0.4 (2.0)	0.03
**Complications**						
Diabetic Retinopathy, n (%)	993 (16.4)	2276 (11.4)	0.15	646 (13.0)	594 (12.0)	0.03
Diabetic macular edema, n (%)	132 (2.2)	292 (1.5)	0.06	76 (1.5)	83 (1.7)	0.01
Stroke / TIA, n (%)	85 (1.4)	258 (1.3)	<0.01	64 (1.3)	59 (1.2)	<0.01
Carotid atherosclerosis, n (%)	1023 (16.9)	3738 (18.7)	0.05	785 (15.8)	847 (17.0)	0.03
Ischemic heart disease, n (%)	587 (9.7)	1833 (9.2)	0.02	425 (8.6)	426 (8.6)	<0.01
Left ventricular hypertrophy, n (%)	409 (6.8)	1353 (6.8)	<0.01	328 (6.6)	323 (6.5)	<0.01
Heart failure, n (%)	162 (2.7)	361 (1.8)	0.06	127 (2.6)	116 (2.3)	0.01
Any site revascolarization, n (%)	394 (6.5)	1276 (6.4)	<0.01	281 (5.7)	297 (6.0)	0.01
Microvascular complications, n (%)	1289 (21.3)	3366 (16.9)	0.12	880 (17.7)	821 (16.5)	0.03
Macrovascular complications, n (%)	1756 (29.0)	5970 (29.9)	0.02	1351 (27.2)	1373 (27.6)	<0.01
Established CVD, n (%)	721 (11.9)	2285 (11.4)	0.01	528 (10.6)	530 (10.7)	<0.01
**Glucose lowering medications**						
Metformin, n (%)	4999 (82.6)	16031 (80.3)	0.06	4277 (86.1)	4269 (85.9)	<0.01
Sulphonylurea / repaglinide, n (%)	507 (8.4)	3809 (19.1)	0.29	505 (10.2)	520 (10.5)	<0.01
DPP-4 inhibitors, n (%)	78 (1.3)	2157 (10.8)	0.34	77 (1.5)	83 (1.7)	<0.01
GLP-1 receptor agonists, n (%)	146 (2.4)	776 (3.9)	0.08	143 (2.9)	125 (2.5)	0.02
Pioglitazone, n (%)	131 (2.2)	948 (4.7)	0.13	127 (2.6)	129 (2.6)	<0.01

Acarbose, n (%)	49 (0.8)	154 (0.8)	<0.01	42 (0.8)	39 (0.8)	<0.01
Bolus insulin, n (%)	1428 (23.6)	379 (1.9)	0.91	346 (7.0)	361 (7.3)	0.01
Basal insulin, n (%)	2399 (39.6)	2879 (14.4)	0.65	1341 (27.0)	1255 (25.3)	0.04
**Other medications**						
Statins, n (%)	3253 (53.7)	10281 (51.5)	0.05	2579 (51.9)	2597 (52.3)	<0.01
Anti-platelet agents, n (%)	2057 (34.0)	6802 (34.1)	<0.01	1576 (31.7)	1553 (31.3)	<0.01
RAS blockers, n (%)	3270 (54.0)	10387 (52.0)	0.04	2624 (52.8)	2578 (51.9)	0.02
Beta blockers, n (%)	1542 (25.5)	4774 (23.9)	0.04	1223 (24.6)	1198 (24.1)	0.01
Calcium channel inhibitors, n (%)	1124 (18.6)	3645 (18.3)	<0.01	903 (18.2)	895 (18.0)	<0.01
Diuretics, n (%)	1564 (25.8)	5255 (26.3)	0.01	1223 (24.6)	1225 (24.7)	<0.01
Anticoagulants, n (%)	94 (1.6)	417 (2.1)	0.04	78 (1.6)	81 (1.6)	<0.01

Microvascular complications included retinopathy, neuropathy, and nephropathy. Macrovascular complications included evidence of atherosclerosis in any arterial site. Established cardiovascular disease (CVD) was defined as myocardial infarction, stroke or transient ischemic attack (TIA), or prior arterial revascularization. RAS, renin angiotensin system. SMD, standardized mean difference.

Table S4. Clinical characteristics of cohorts of patients in the analysis excluding GLP-1RA as comparators. Data are presented for patients who initiated dapagliflozin or comparators other than GLP-1RA before and after propensity score matching. Continuous variables are presented as mean (standard deviation) and median (IQR). Categorical variables are presented as number (percentage).

	Before PSM			After PSM		
	**Dapagliflozin**	**Comparators**	**SMD**	**Dapagliflozin**	**Comparators**	**SMD**
**Number**	7294	20533		5609	5609	
**Demographics**						
Sex male, n (%)	4388 (60.2)	12599 (61.4)	0.02	3435 (61.2)	3507 (62.5)	0.03
Age, years	60.9 (9.0)	64.5 (8.4)	0.42	61.2 (9.0)	61.3 (9.6)	0.02
Diabetes duration, years	11.6 (8.4)	10.7 (7.9)	0.11	10.3 (7.9)	10.4 (8.2)	<0.01
**Anthropometrics**						
Weight, kg	88.7 (18.4)	80.0 (15.9)	0.52	86.5 (17.3)	85.9 (17.5)	0.03
Height, cm	167.1 (9.7)	166.2 (9.6)	0.09	167.1 (9.6)	167.3 (9.7)	0.02
Body mass index, kg/m2	31.7 (5.8)	29.0 (5.1)	0.51	30.9 (5.5)	30.7 (5.7)	0.04
Waist, cm	109.2 (13.4)	102.9 (12.3)	0.49	107.2 (12.6)	106.3 (12.8)	0.07
**Risk factors and laboratory**						
Systolic blood pressure, mm Hg	137.9 (19.0)	136.0 (18.5)	0.10	137.0 (18.8)	136.9 (18.5)	<0.01
Diastolic blood pressure, mm Hg	79.3 (10.3)	77.6 (9.8)	0.17	79.3 (10.2)	79.2 (10.1)	<0.01
Fasting plasma glucose, mg/dl	173.9 (57.0)	158.8 (48.0)	0.30	167.8 (52.3)	168.3 (53.8)	0.01
HbA1c, %	8.4 (1.5)	7.8 (1.2)	0.46	8.2 (1.4)	8.2 (1.4)	<0.01
Total cholesterol, mg/dl	174.8 (42.8)	172.8 (42.4)	0.05	174.3 (41.9)	174.3 (43.3)	<0.01
HDL cholesterol, mg/dl	46.6 (14.3)	49.0 (15.5)	0.16	47.2 (14.4)	46.9 (14.6)	0.02
LDL cholesterol, mg/dl	95.4 (35.7)	94.6 (35.7)	0.02	95.1 (35.3)	95.5 (36.4)	0.01
Triglycerides, mg/dl	171.6 (132.4)	152.1 (106.0)	0.17	167.7 (127.4)	166.3 (132.0)	0.01
eGFR (ml/min/1.73 m^2)	87.9 (16.5)	79.6 (21.4)	0.41	87.8 (16.6)	87.7 (17.8)	<0.01
Albumin excretion rate, mg/g	73.9 (589.9)	74.0 (678.9)	<0.01	58.7 (292.8)	66.2 (621.2)	0.02
eGFR slope (ml/min/1.73 m^2/year)	-0.6 (2.4)	-1.12 (2.4)	0.21	-0.6 (2.2)	-0.7 (2.2)	0.05
**Complications**						
Chronic kidney disease, n (%)	1455 (19.9)	5903 (28.7)	0.20	1074 (19.1)	1116 (19.9)	0.02
eGFR <60 ml/min/1.73 m^2, n (%)	385 (5.3)	3727 (18.2)	0.37	333 (5.9)	329 (5.9)	<0.01
UACR >30 mg/g, n (%)	1176 (16.1)	3035 (14.8)	0.04	828 (14.8)	867 (15.5)	0.02
Diabetic Retinopathy, n (%)	1341 (18.4)	2738 (13.3)	0.14	793 (14.1)	792 (14.1)	<0.01
Diabetic macular edema, n (%)	192 (2.6)	380 (1.9)	0.06	113 (2.0)	101 (1.8)	0.02
Stroke / TIA, n (%)	103 (1.4)	345 (1.7)	0.02	73 (1.3)	80 (1.4)	0.01
Carotid atherosclerosis, n (%)	1337 (18.3)	4454 (21.7)	0.08	985 (17.6)	1004 (17.9)	<0.01
Ischemic heart disease, n (%)	767 (10.5)	2268 (11.0)	0.02	545 (9.7)	554 (9.9)	<0.01
Left ventricular hypertrophy, n (%)	558 (7.7)	1662 (8.1)	0.02	397 (7.1)	406 (7.2)	<0.01
Heart failure, n (%)	224 (3.1)	515 (2.5)	0.04	154 (2.7)	136 (2.4)	0.02
Any site revascolarization, n (%)	500 (6.9)	1572 (7.7)	0.03	354 (6.3)	348 (6.2)	<0.01
Microvascular complications, n (%)	2688 (36.9)	8445 (41.1)	0.09	1849 (33.0)	1876 (33.4)	0.01
Macrovascular complications, n (%)	2268 (31.1)	7096 (34.6)	0.07	1644 (29.3)	1657 (29.5)	<0.01
Established CVD, n (%)	933 (12.8)	2853 (13.9)	0.03	660 (11.8)	678 (12.1)	<0.01
**Glucose lowering medications**						
Metformin, n (%)	5932 (81.3)	14923 (72.7)	0.20	4731 (84.3)	4714 (84.0)	<0.01
Sulphonylurea / repaglinide, n (%)	570 (7.8)	4384 (21.4)	0.36	564 (10.1)	582 (10.4)	0.01

DPP-4 inhibitors, n (%)	108 (1.5)	3124 (15.2)	0.44	107 (1.9)	115 (2.1)	0.01
GLP-1 receptor agonists, n (%)	0 (0.0)	0 (0.0)	<0.01	0 (0.0)	0 (0.0)	<0.01
Pioglitazone, n (%)	134 (1.8)	845 (4.1)	0.12	126 (2.2)	132 (2.4)	<0.01
Acarbose, n (%)	57 (0.8)	182 (0.9)	0.01	46 (0.8)	43 (0.8)	<0.01
Bolus insulin, n (%)	1927 (26.4)	487 (2.4)	0.92	446 (8.0)	424 (7.6)	0.01
Basal insulin, n (%)	3068 (42.1)	2859 (13.9)	0.72	1483 (26.4)	1451 (25.9)	0.01
**Other medications**						
Statins, n (%)	4009 (55.0)	11032 (53.7)	0.02	2992 (53.3)	3033 (54.1)	0.01
Anti-platelet agents, n (%)	2630 (36.1)	7813 (38.1)	0.04	1908 (34.0)	1929 (34.4)	<0.01
RAS blockers, n (%)	4156 (57.0)	11380 (55.4)	0.03	3089 (55.1)	3133 (55.9)	0.02
Beta blockers, n (%)	1953 (26.8)	5364 (26.1)	0.01	1443 (25.7)	1458 (26.0)	<0.01
Calcium channel inhibitors, n (%)	1497 (20.5)	4308 (21.0)	0.01	1126 (20.1)	1118 (19.9)	<0.01
Diuretics, n (%)	2019 (27.7)	6167 (30.0)	0.05	1475 (26.3)	1492 (26.6)	<0.01
Anticoagulants, n (%)	137 (1.9)	675 (3.3)	0.08	108 (1.9)	102 (1.8)	<0.01

Microvascular complications included retinopathy, neuropathy, and nephropathy. Macrovascular complications included evidence of atherosclerosis in any arterial site. Established cardiovascular disease (CVD) was defined as myocardial infarction, stroke or transient ischemic attack (TIA), or prior arterial revascularization. RAS, renin angiotensin system. SMD, standardized mean difference.

Figure S1. Comparison of the paired and unpaired analytical approach. a) Primary endpoint: change in eGFR over time (positive values indicate higher eGFR in the dapagliflozin group). b) Main secondary categorical endpoint: composite kidney outcome (HR<1 indicates lower rates in the dapagliflozin group).

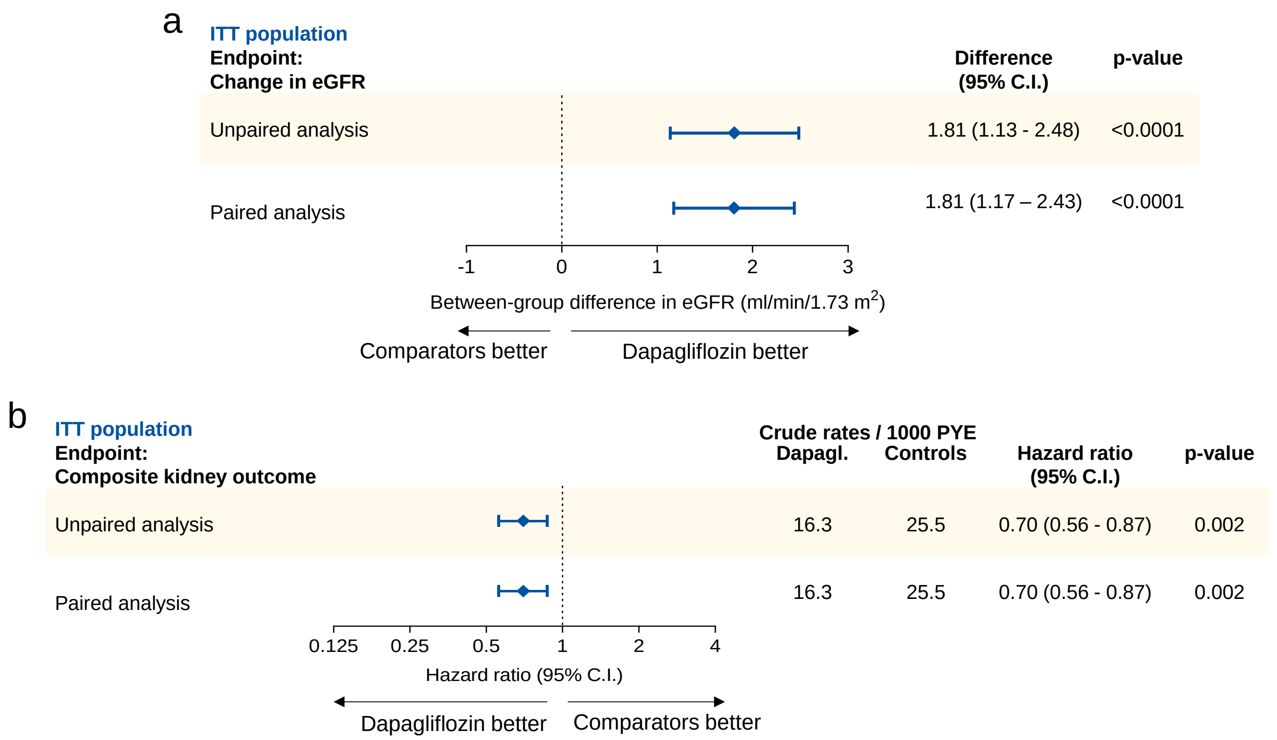

Figure S2. Plot of pre- and post-index date eGFR values. Data are modelled with the mixed model for repeated measures. Numbers in the table below the graph indicate the number of patients contributing to those specific time points. The median (IQR) number of pre-index date eGFR values per patient was 6 (3-11). The median (IQR) collection time of pre-index date eGFR was 4.8 (1.5-9.0) years.

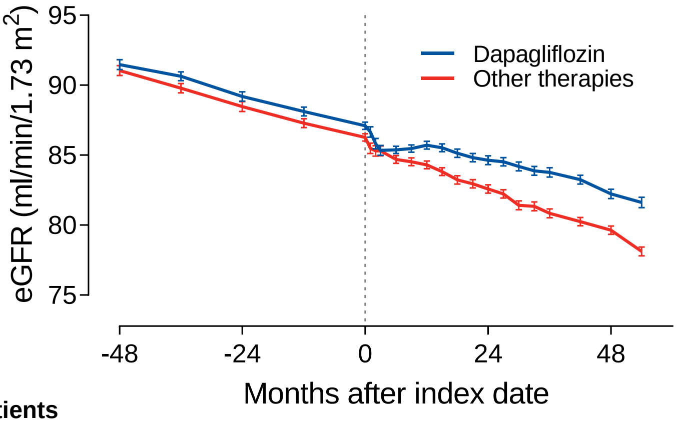

**No. of patients**					
Dapagliflozin	1640	1475	5829	1418	1058
Comparators	1464	1157	5636	1530	1486

Figure S3. Results of the on-treatment analyses. Changes in eGFR (a) and albuminuria (urinary albumin creatinine ratio, UACR, b) in initiators of dapagliflozin or comparators are shown with patients censored at discontinuation of index drug.

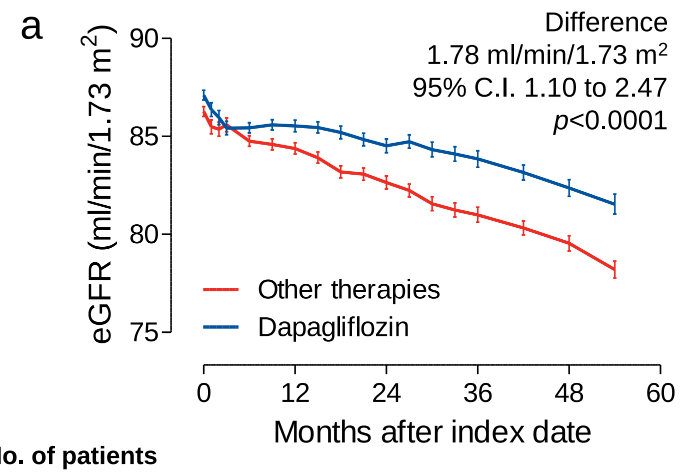

No. of patients

	0	12	24	36	48	60
Dapagliflozin	4548	1747	1077	616	687	431*
Comparators	4211	1827	1250	870	1016	750*

**54 months*

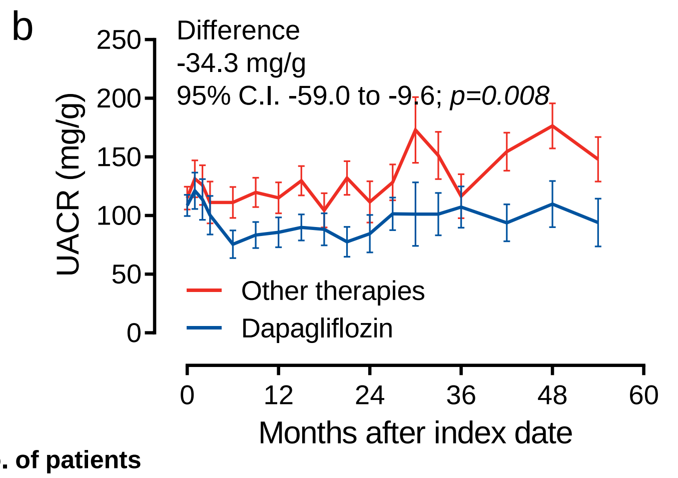

No. of patients

	0	12	24	36	48	60
Dapagliflozin	2622	602	370	254	258	164*
Comparators	2533	655	408	283	376	282*

**54 months*

Figure S4. Main results of the comparison between initiators of SGLT2 inhibitors and initiators of comparators. Changes over time in eGFR (a) and urinary albumin excretion rate (UACR, b), in the groups of patients who initiated any SGLT2 inhibitors (SGLT2i) or comparators. Panel c) shows the hazard ratios (HR) and 95% confidence intervals (C.I.) for categorical eGFR-based endpoints in the SGLT2i versus comparators group. Cumulative proportion of patients with an event (from 0 to 1) from Cox models are presented for creatinine doubling (≥57% eGFR reduction, d) and end-stage kidney disease (ESKD, e).

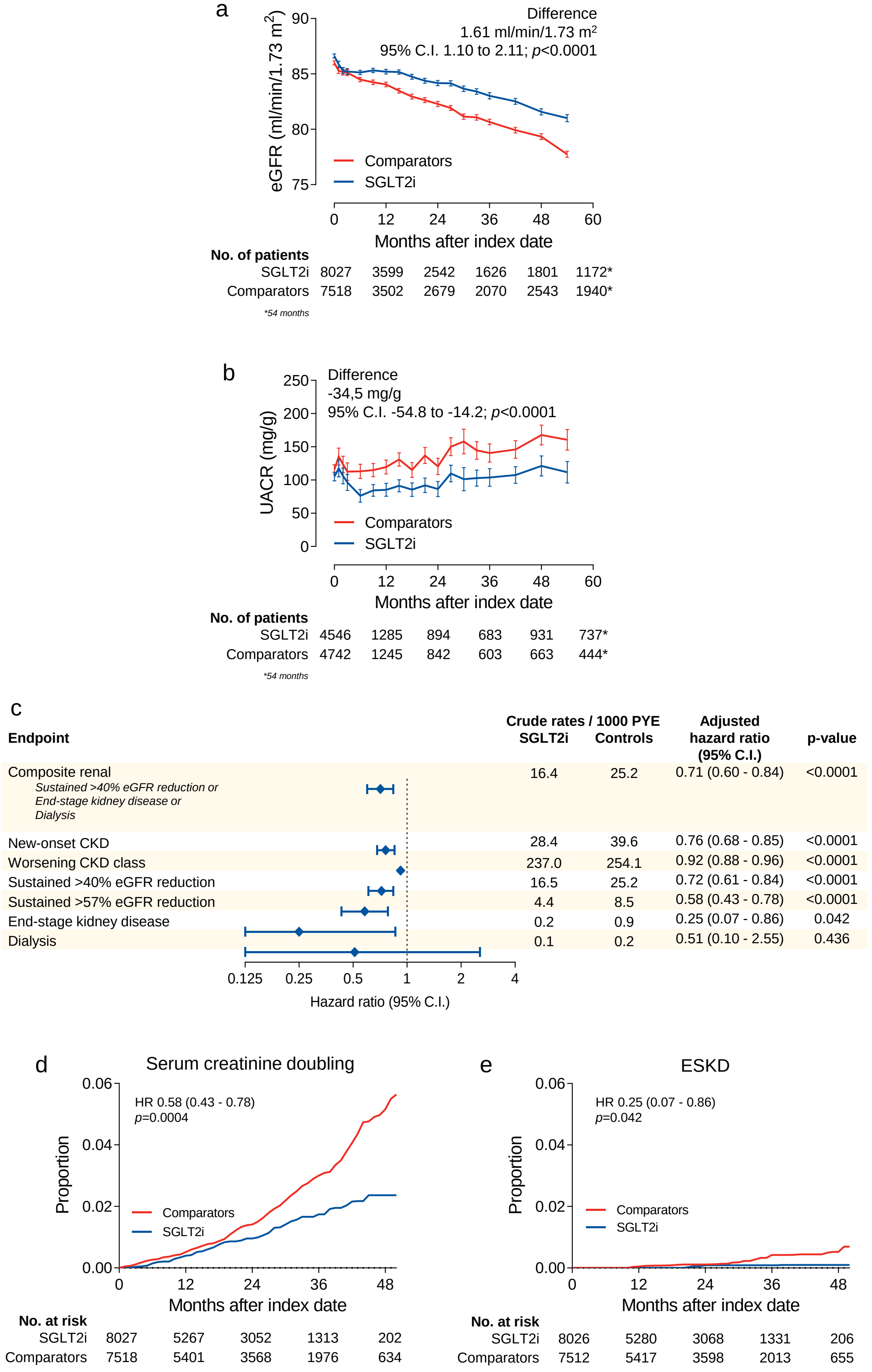

Figure S5. **Main results of the cohort of patients without baseline CKD.** Changes over time in eGFR (a) and urinary albumin excretion rate (UACR, b), in the groups of patients who initiated any dapagliflozin or comparators. Panel c) shows the hazard ratios (HR) and 95% confidence intervals (C.I.) for categorical eGFR-based endpoints in the dapagliflozin versus comparators group. Cumulative proportion of patients with a event (from 0 to 1) from Cox models are presented for confirmed new-onset CKD (d) and ≥40% loss of kidney function (e).

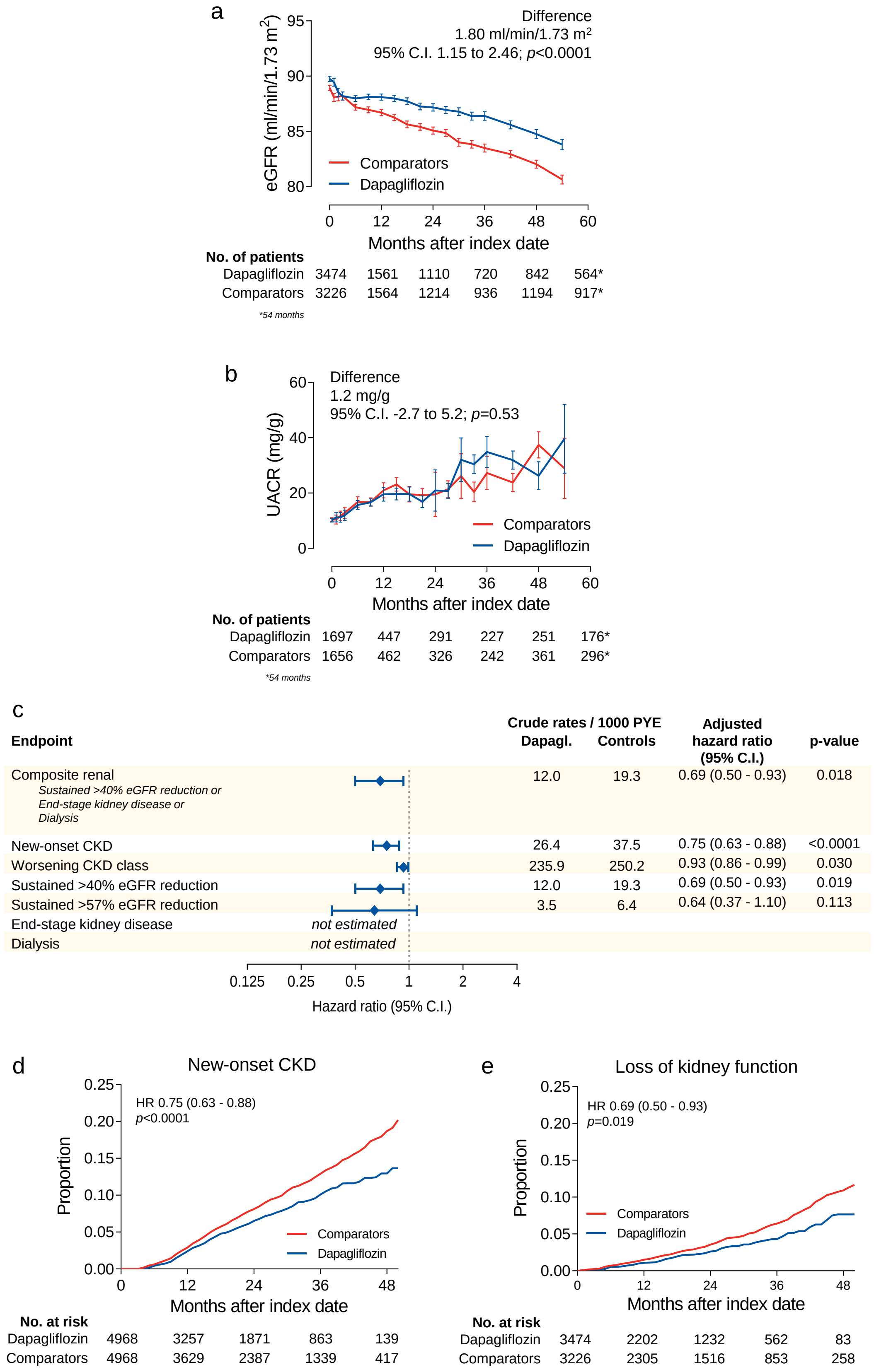

Figure S6. Main results of the cohort of patients without GLP-1RA new-users in the comparator group. Changes over time in eGFR (a) and urinary albumin excretion rate (UACR, b), in the groups of patients who initiated any dapagliflozin or comparators. c) Hazard ratios (HR) and 95% confidence intervals (C.I.) for categorical eGFR-based endpoints in the dapagliflozin versus comparators group. Cumulative proportion (from 0 to 1) of patients with an event from Cox models are presented for the composite kidney outcome (e) and ≥57% loss of kidney function, equal to serum creatinine doubling (e).

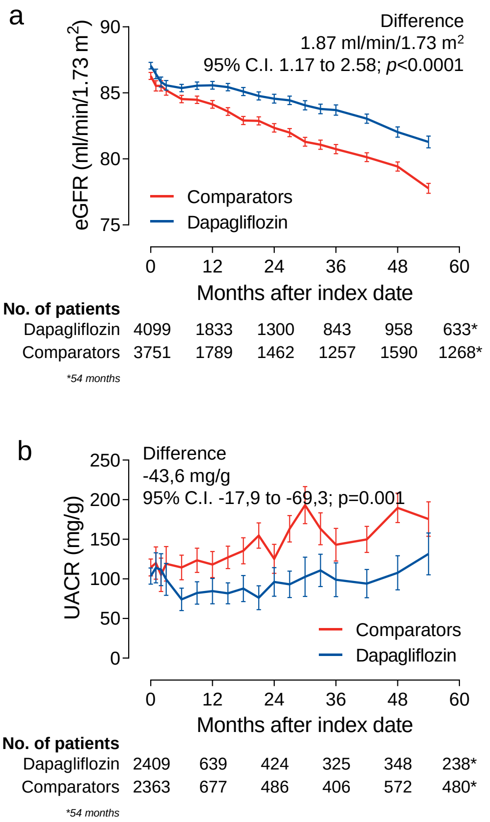

c

Endpoint	Crude rates / 1000 PYE Dapagl.	Controls	Adjusted hazard ratio (95% C.I.)	p-value
Composite renal *Sustained >40% eGFR reduction or End-stage kidney disease or Dialysis*	15.8	26.5	0.68 (0.54 - 0.85)	0.001
New-onset CKD	28.2	40.4	0.76 (0.64 - 0.91)	0.005
Worsening CKD class	236.9	243.7	0.95 (0.89 - 1.01)	0.110
Sustained >40% eGFR reduction	15.8	26.5	0.68 (0.54 - 0.85)	0.001
Sustained >57% eGFR reduction	4.6	9.1	0.61 (0.41 - 0.93)	0.023
End-stage kidney disease	0.1	1.2		
Dialysis	0.2	0.4	0.46 (0.09 - 2.48)	0.400

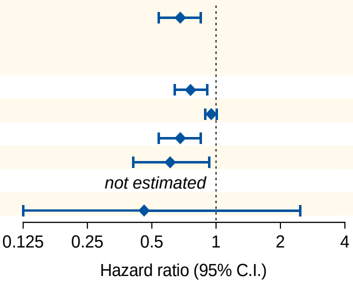

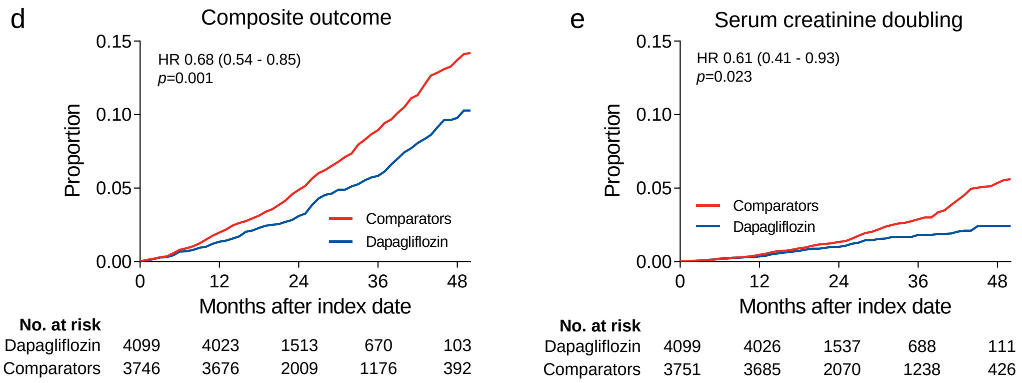

Supplement: Online Data Supplement [file mmc1.pdf]
